# Supplementary material for: Controlling gene expression with deep generative design of regulatory DNA
Source: Nat Commun. 2022 Aug 30;13:5099. doi: 10.1038/s41467-022-32818-8 (PMC9427793; doi:10.1038/s41467-022-32818-8)
Supplement: Supplementary file 1 — Supplementary Information [file 41467_2022_32818_MOESM1_ESM.pdf]

Controlling gene expression with deep generative design of regulatory DNA

Jan Zrimec[1,2*#], Xiaozhi Fu[1*], Azam Sheikh Muhammad[3], Christos Skrekas[1], Vykintas Jauniskis[1,4], Nora K. Speicher[3], Christoph S. Börlin[1,5], Vilhelm Verendel[3], Morteza Haghir Chehreghani[3], Devdatt Dubhashi[3], Verena Siewers[1], Florian David[1], Jens Nielsen[1,5], Aleksej Zelezniak[1,6,7#]

1 - Department of Biology and Biological Engineering, Chalmers University of Technology, Kemivägen 10, SE41296 Gothenburg, Sweden

2 - Department of Biotechnology and Systems Biology, National Institute of Biology, Večna pot 111, SI1000 Ljubljana, Slovenia

3 - Department of Computer Science and Engineering, Chalmers University of Technology, Rännvägen 6, SE41296 Gothenburg, Sweden

4 - Biomatter Designs, Zirmunu st. 139A, LT09120 Vilnius, Lithuania

5 - BioInnovation Institute, Ole Maaloes Vej 3, DK2200 Copenhagen N, Denmark

6 - Institute of Biotechnology, Life Sciences Centre, Vilnius University, Sauletekio al. 7, LT10257 Vilnius, Lithuania

7 - Randall Centre for Cell & Molecular Biophysics, King's College London, New Hunt's House, Guy's Campus, SE1 1UL London, UK

[#] corresponding authors (email: aleksej.zelezniak@chalmers.se, jan.zrimec@nib.si)

* These authors contributed equally.

Table of contents

Supplementary Figures	…	p.2 - p.28
Supplementary Tables	…	p.29 - p.55
Supplementary References	…	p.56 - p.57

Supplementary Figures

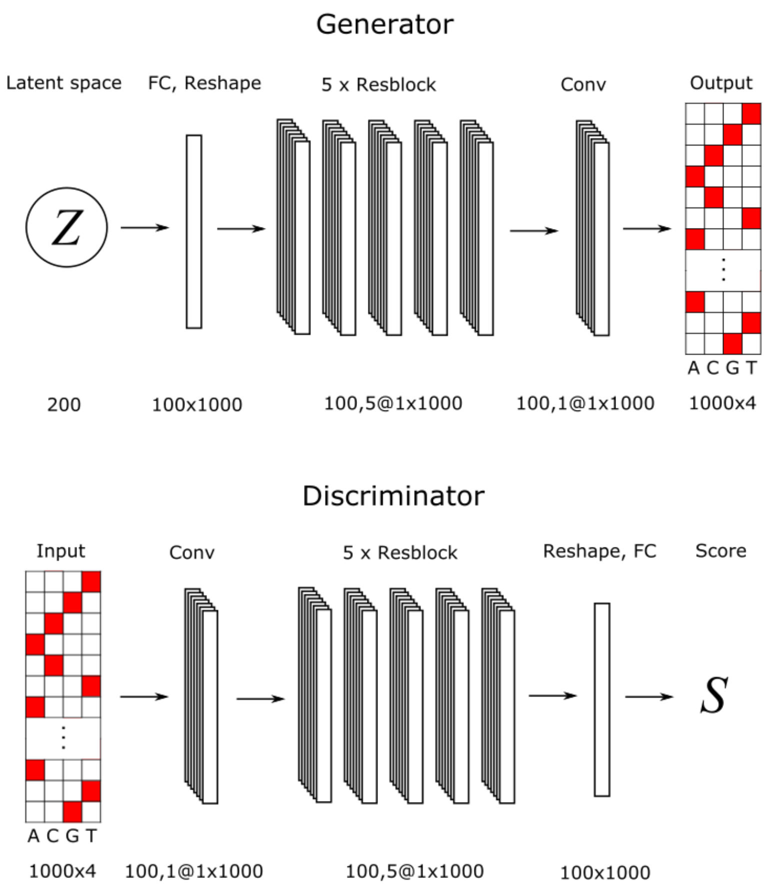

Supplementary Figure 1. Overview of generative adversarial network (GAN) approach and its deep neural network architectures. Red squares denote input values of 1 and white squares zeros.

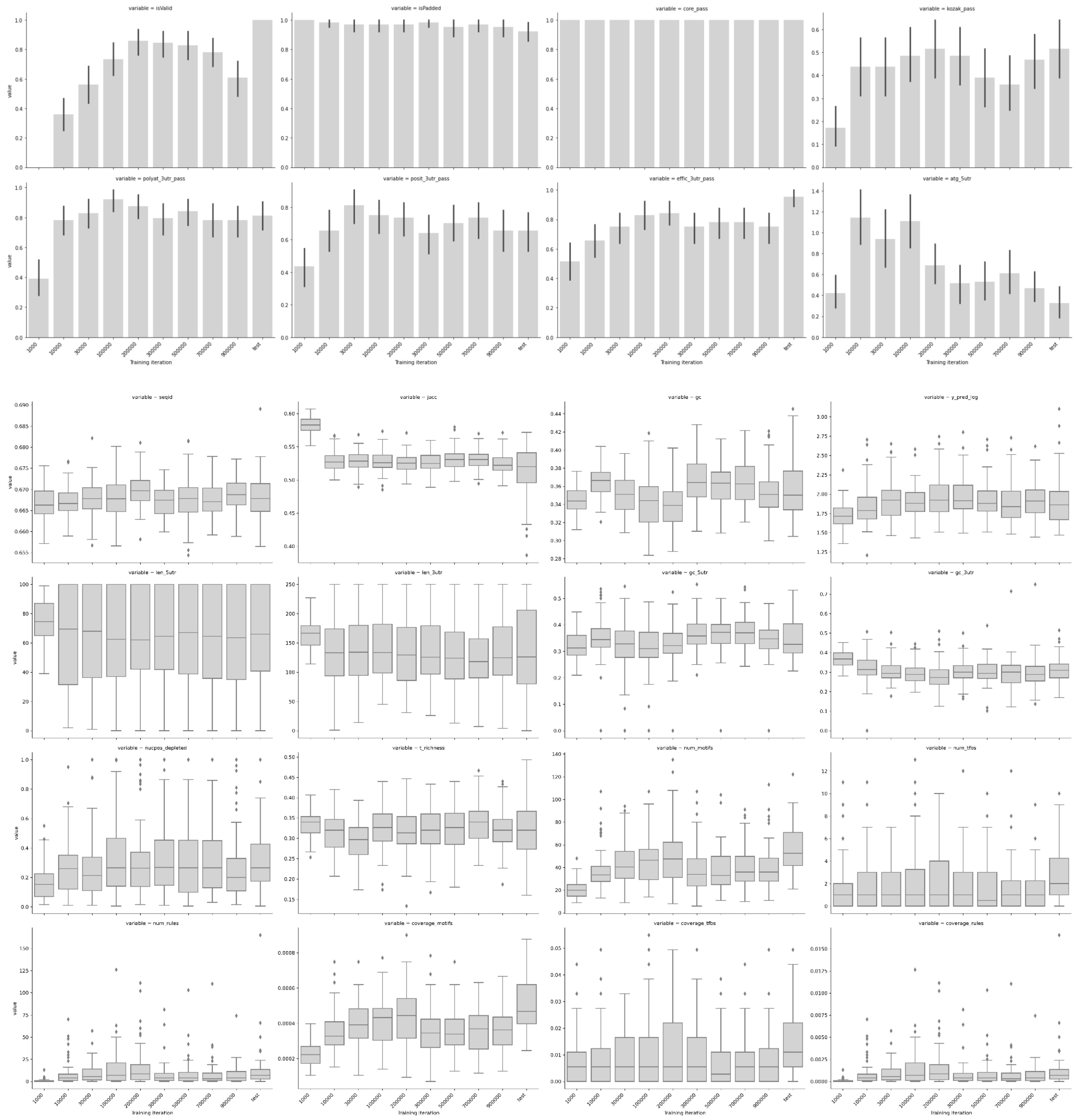

Supplementary Figure 2. Computed sequence properties (see Supplementary Table 3) of generated sequence variants sampled from the generative model after different amounts of training iterations (*n* = 64 each). For bar plots, error bars represent 95% confidence intervals. For box plots, Boxes denote interquartile (IQR) ranges, centres mark medians and whiskers extend to 1.5 IQR from the quartiles.

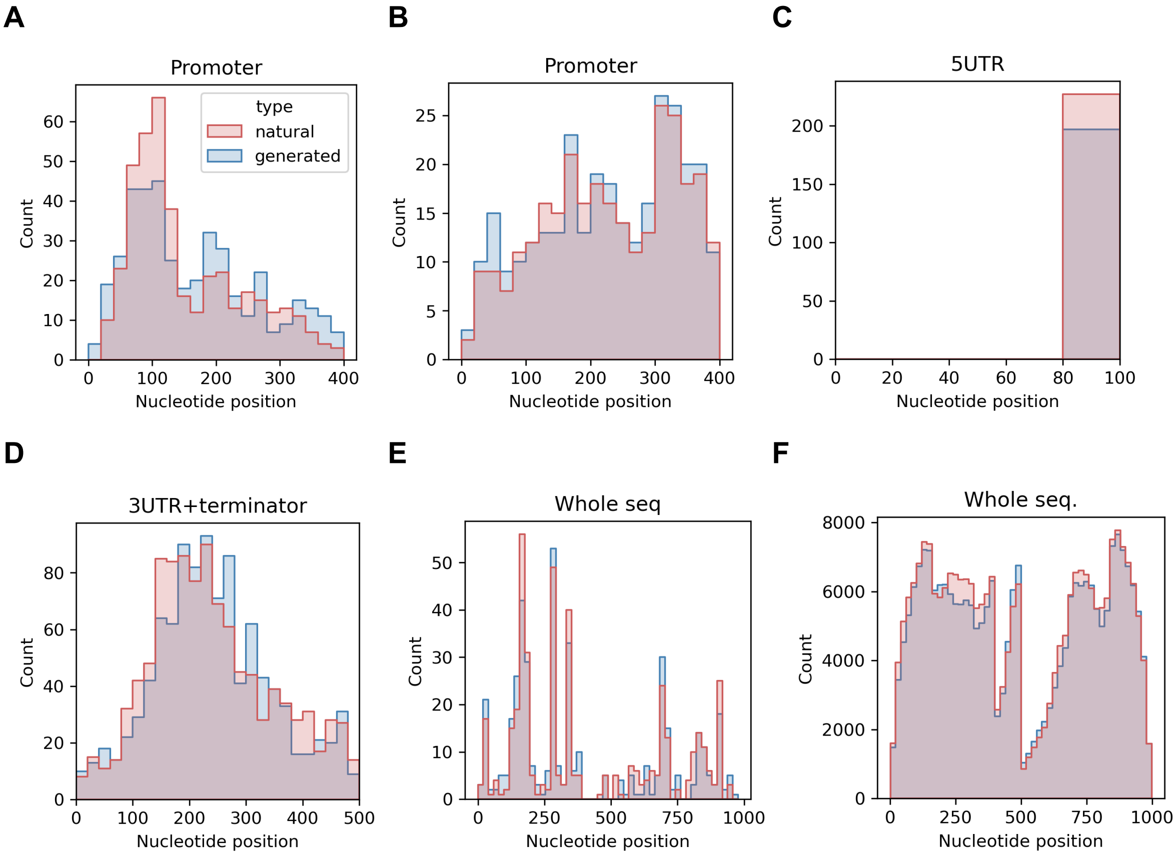

Supplementary Figure 3. Comparison of positional distributions of DNA regulatory grammar elements in sets of generated (red) and natural test (blue) sequences, including (A) transcription factor binding sites (TFBS) [1] and (B) TATA core promoter elements [2,3] in promoters, (C) Kozak sequences [4,5] in 5' UTRs, (D) termination related motifs (positioning, efficiency and poly-AT motifs) [6,7] in 3' UTRs and terminators, and (E) deep learning-uncovered expression-related motifs and motif association rules [8] as well as (F) nucleosome depletion [9,10] across all regions, with the nucleotide positions binned into 20 bp intervals. Relation of the given x axis to the gene is depicted in Figure 1A: promoters are located -400 bp upstream to the transcription start site, 5' UTR -100 bp upstream to the Start codon, 3' UTRs+terminators -250 bp upstream to +250 bp downstream of the transcription termination site.

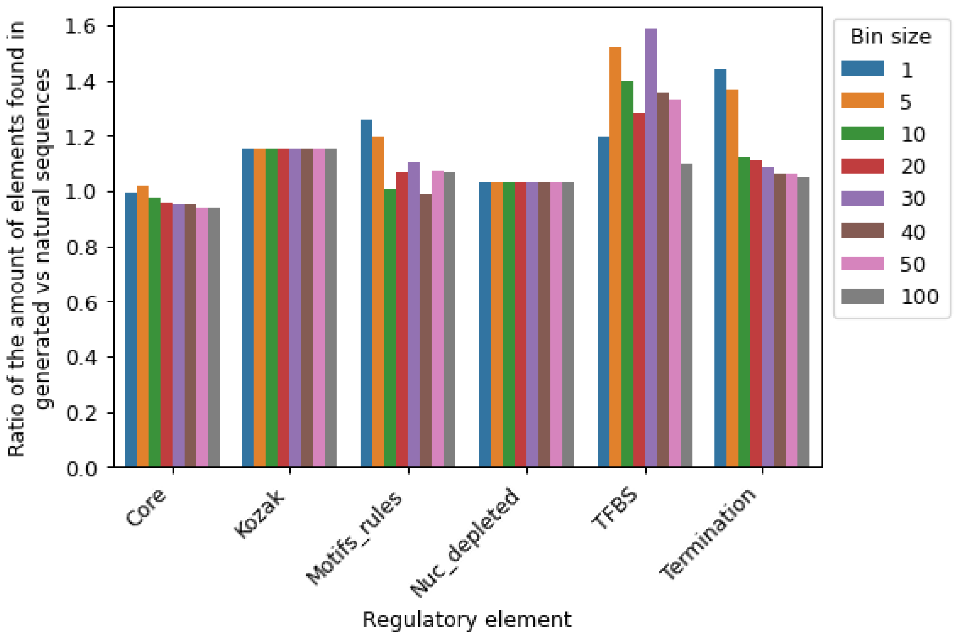

Supplementary Figure 4. Analysis of the overall number of DNA regulatory motifs and positions in the generated sequences compared to the amounts found in natural test sequences, when the corresponding locations were binned into different sized intervals (see Figure 2A,B regarding the description of the analyzed regulatory elements). Bin sizes are marked with colors: 1 blue, 5 orange, 10 green, 20 red, 30 violet, 40 brown, 50 pink and 100 gray.

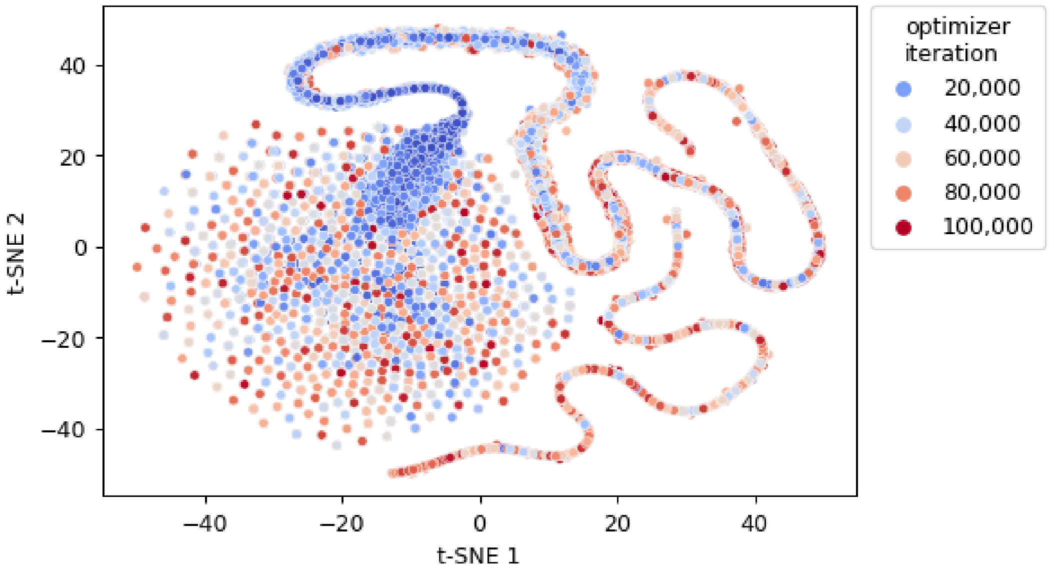

Supplementary Figure 5. T-distributed stochastic neighbour embedding (t-SNE) dimensionality reduction [11] over the latent space vectors of generated sequence variants with the predictor-guided optimization approach, merging the results of both maximization and minimization of gene expression and marked by optimizer iterations. The progression of optimizer iterations from 0 to 100,000 is marked with progression from blue to red, respectively.

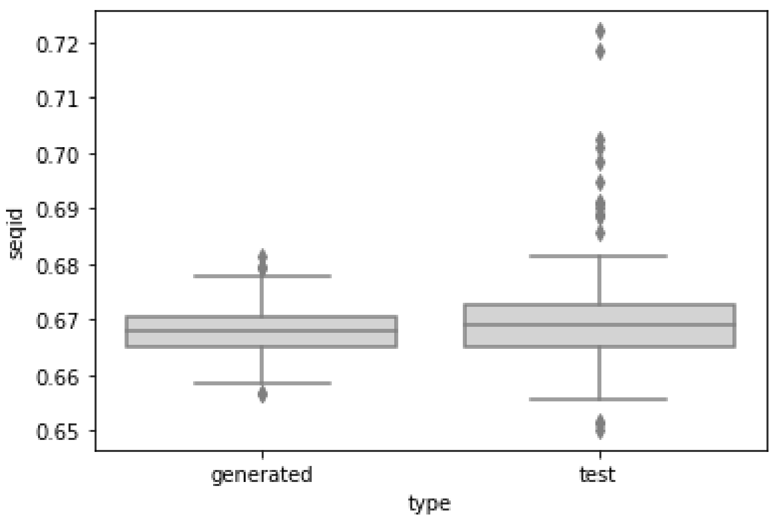

Supplementary Figure 6. Sequence identity of generated and natural test set sequences (*n* = 425 each) versus their closest matching sequences in the training dataset. Boxes denote interquartile (IQR) ranges, centres mark medians and whiskers extend to 1.5 IQR from the quartiles.

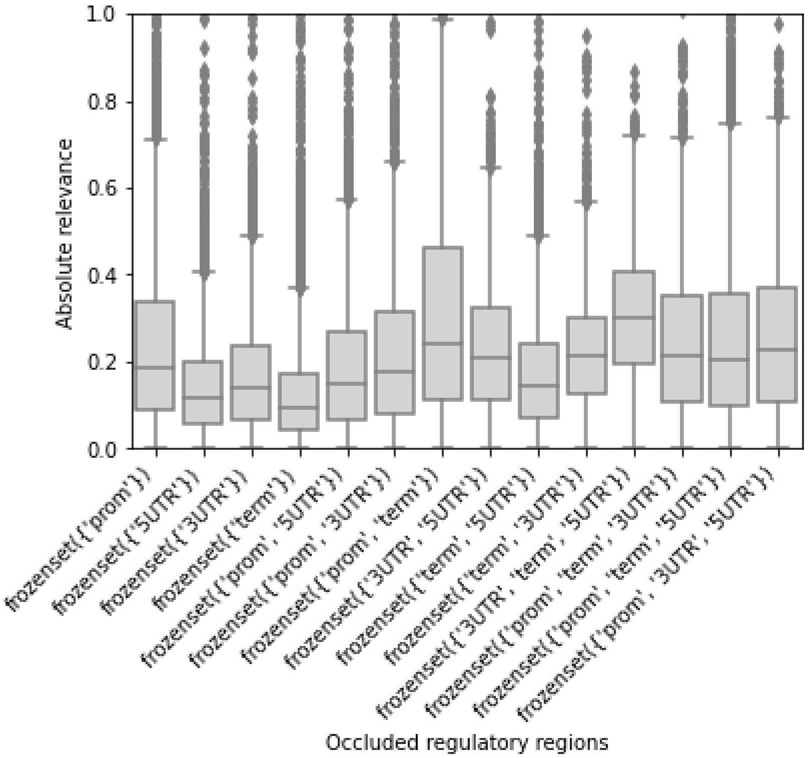

Supplementary Figure 7. Analysis of the relevance of different regulatory region combinations. Combinations of regions were occluded and absolute relevance scores were computed (see Methods M3) using the training dataset (*n* = 4238 each, Methods M1). Median absolute relevance levels were 0.13, 0.19 and 0.24 with a number of 1, 2 or 3 occluded regions, respectively. The results suggest that the whole gene regulatory structure is important for fine-tuning gene expression, as each region carries information that contributes to prediction of expression levels [12–17], with region combinations jointly controlling gene expression [8,18]. Boxes denote interquartile (IQR) ranges, centres mark medians and whiskers extend to 1.5 IQR from the quartiles.

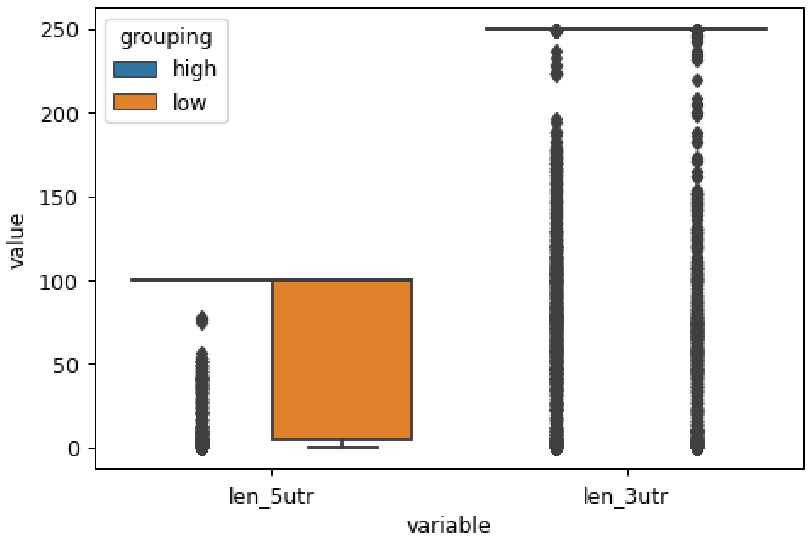

Supplementary Figure 8. Distributions of UTR lengths in high (blue) vs low (orange) expression sequences (n = 10,000 each). Boxes denote interquartile (IQR) ranges, centres mark medians and whiskers extend to 1.5 IQR from the quartiles.

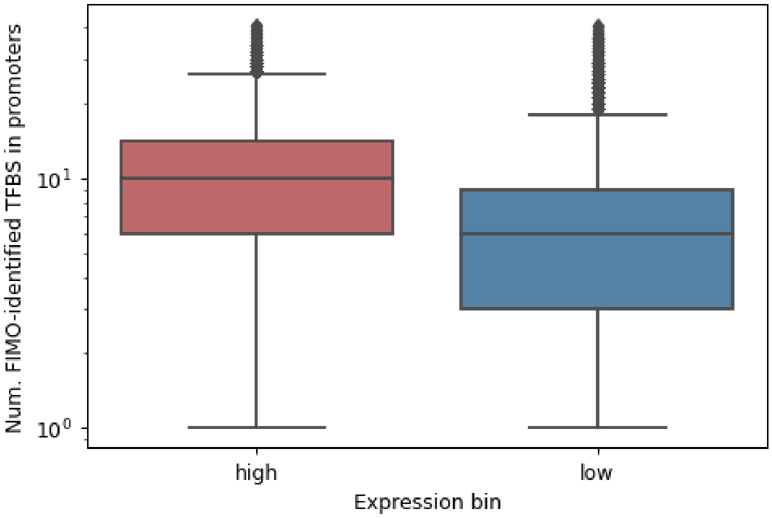

Supplementary Figure 9. Distributions of TFBS identified in the promoter regions of 20,000 generated sequences corresponding to either high (red) or low (blue) expression levels (n = 10,000 each), using the algorithm FIMO [19] and a q-value cutoff of 0.05. Boxes denote interquartile (IQR) ranges, centres mark medians and whiskers extend to 1.5 IQR from the quartiles.

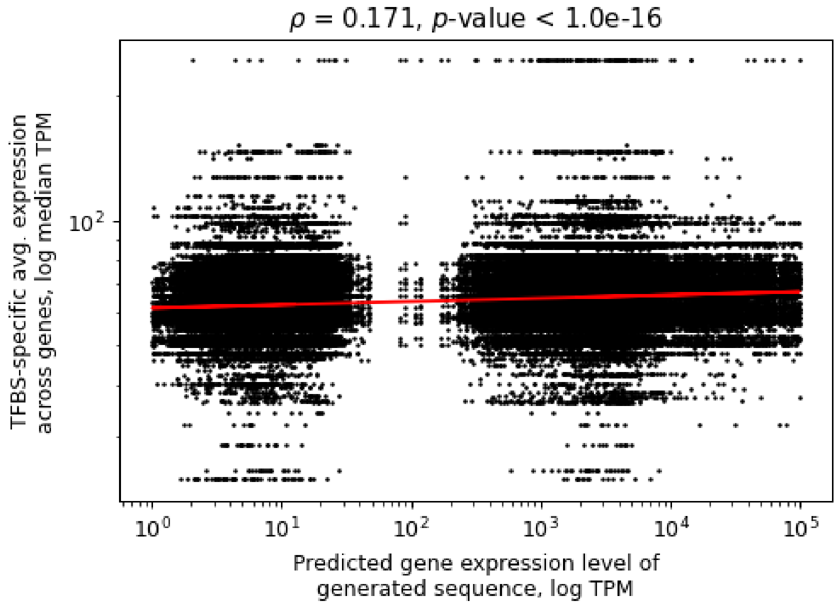

Supplementary Figure 10. Correlation analysis between median expression levels of natural genes, where a TFBS was found, and the predicted expression level of the generated sequence that carries the specific TFBS. Red line denotes least squares fit. The T-test was used for significance testing.

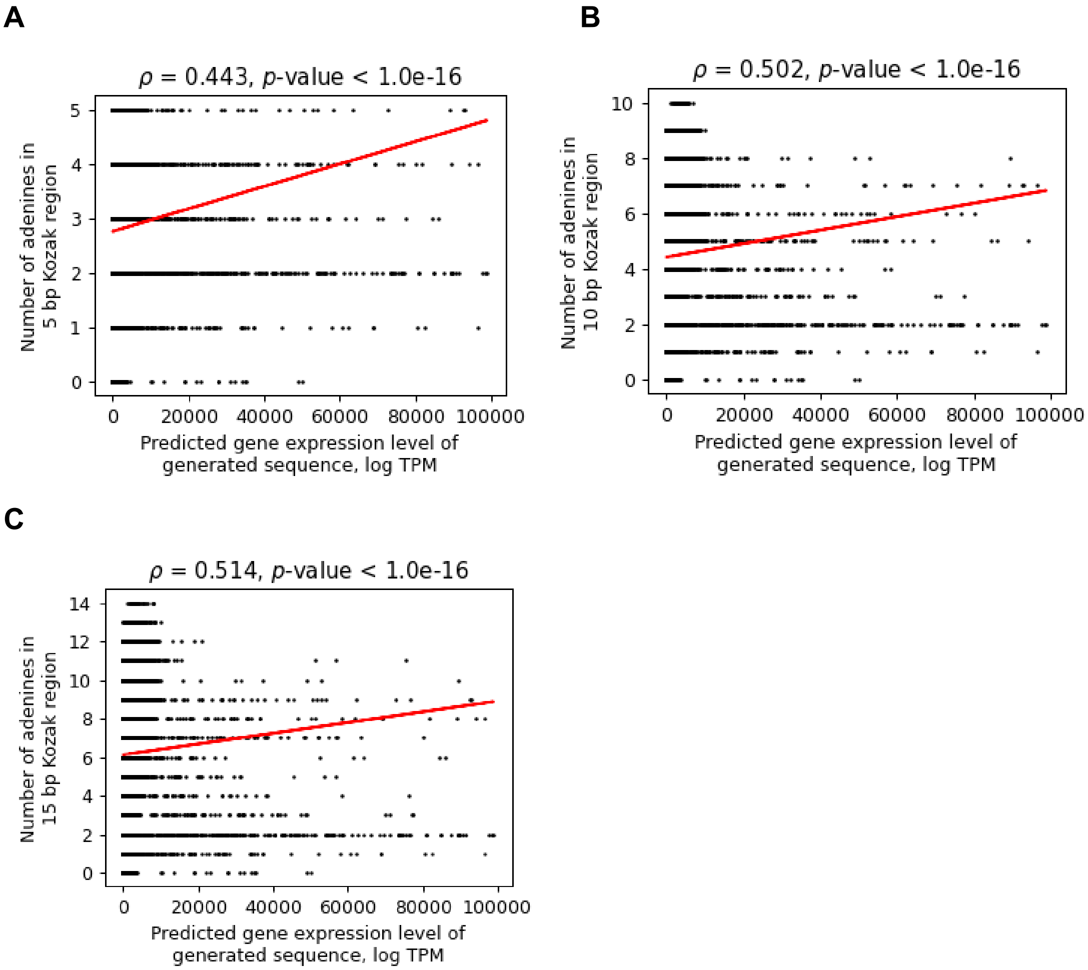

Supplementary Figure 11. Correlation analysis between the number of adenines in the (A) 5, (B) 10 and (C) 15 bp region upstream of the start codon, respectively, and predicted expression levels of the generated sequences. Red lines denote least squares fit. T-test was used.

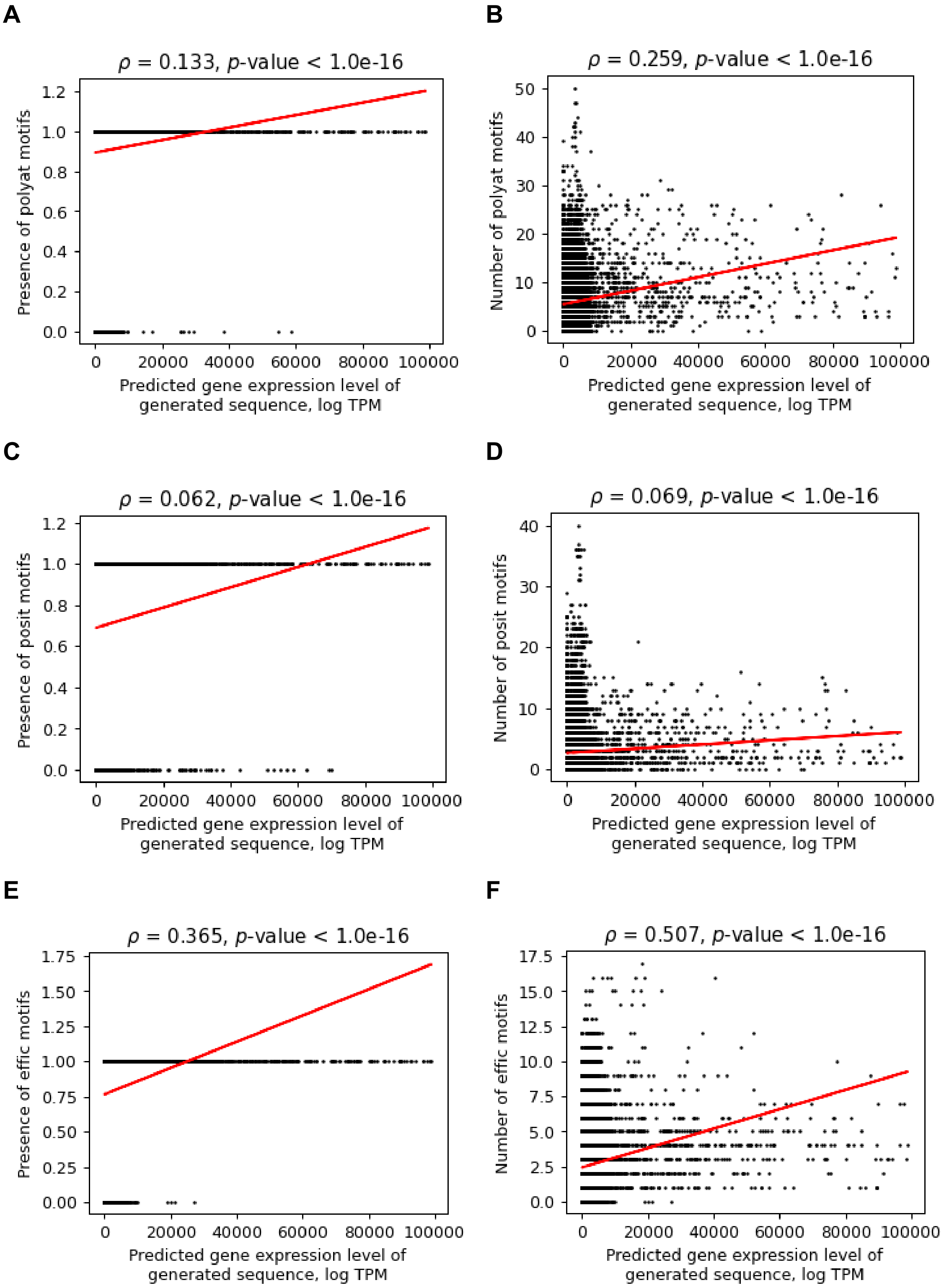
A
ρ = 0.133, p-value < 1.0e-16
Presence of polyat motifs
Predicted gene expression level of generated sequence, log TPM
B
ρ = 0.259, p-value < 1.0e-16
Number of polyat motifs
Predicted gene expression level of generated sequence, log TPM
C
ρ = 0.062, p-value < 1.0e-16
Presence of posit motifs
Predicted gene expression level of generated sequence, log TPM
D
ρ = 0.069, p-value < 1.0e-16
Number of posit motifs
Predicted gene expression level of generated sequence, log TPM
E
ρ = 0.365, p-value < 1.0e-16
Presence of effic motifs
Predicted gene expression level of generated sequence, log TPM
F
ρ = 0.507, p-value < 1.0e-16
Number of effic motifs
Predicted gene expression level of generated sequence, log TPM

Supplementary Figure 12. Correlation analysis between the (A,C,E) presence and (B,D,F) number of (A,B) poly-A/T, (C,D) positioning and (E,F) efficiency motifs [6,16,20] versus predicted expression levels of the generated sequences. Red lines denote least squares fit. T-test was used.

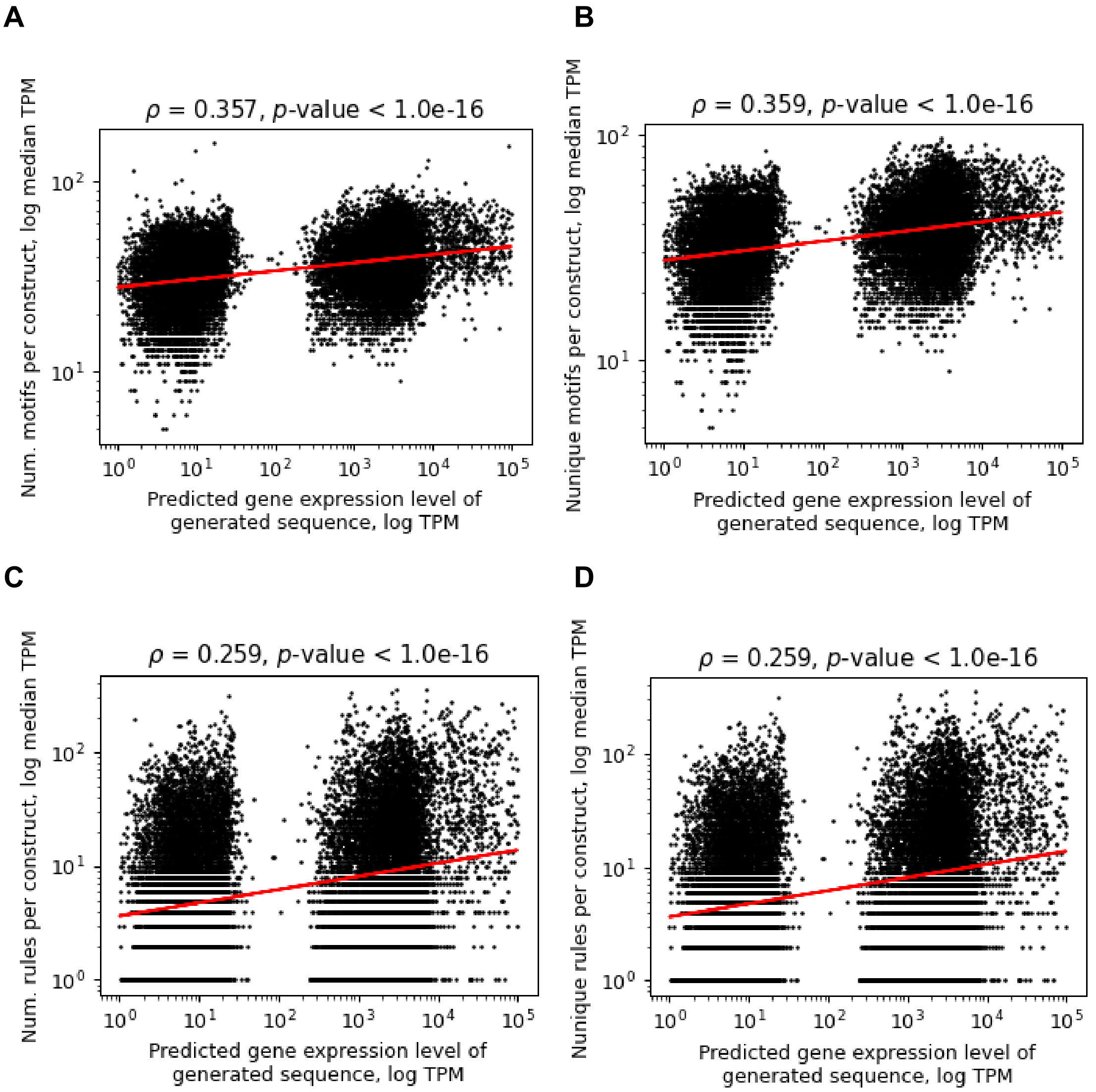

Supplementary Figure 13. Correlation analysis between the number of (A,C,E) all and (B,D,F) unique (A,B) deep-learning uncovered expression-related motifs and (C,D) motif association rules [8] versus predicted expression levels of the generated sequences. Red line denotes least squares fit. T-test was used.

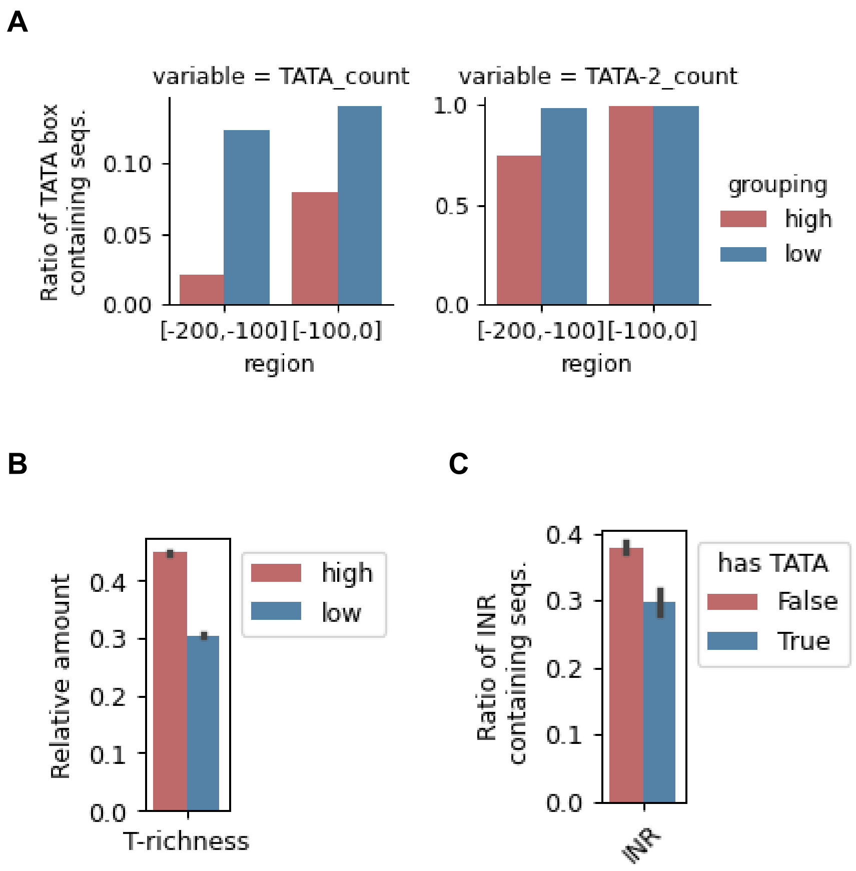

Supplementary Figure 14. Analysis of the core promoter region. (A) Proportion of exact TATA and TATA-like (2 mutations from the consensus 5'-TATAWAWR-3') [2,21] motif-carrying generated sequences in the high (red) and low (blue) expression bins (Figure 4A) across the distal and proximal parts of the core promoter region (Figure 4C). (B) Relative amount of thymines (T-richness) in the [-75, TSS] region [3] of the high (red) and low (blue) expression sequences (*n* = 10,000 each). (C) Proportion of sequences containing the mammalian-type INR motif in the [-30, TSS] region [22] in relation to them also carrying a TATA box (*n* = 16,386 and 3614 with False and True subsets, respectively, marked red and blue, respectively). Error bars in (B, C) represent 95% confidence intervals.

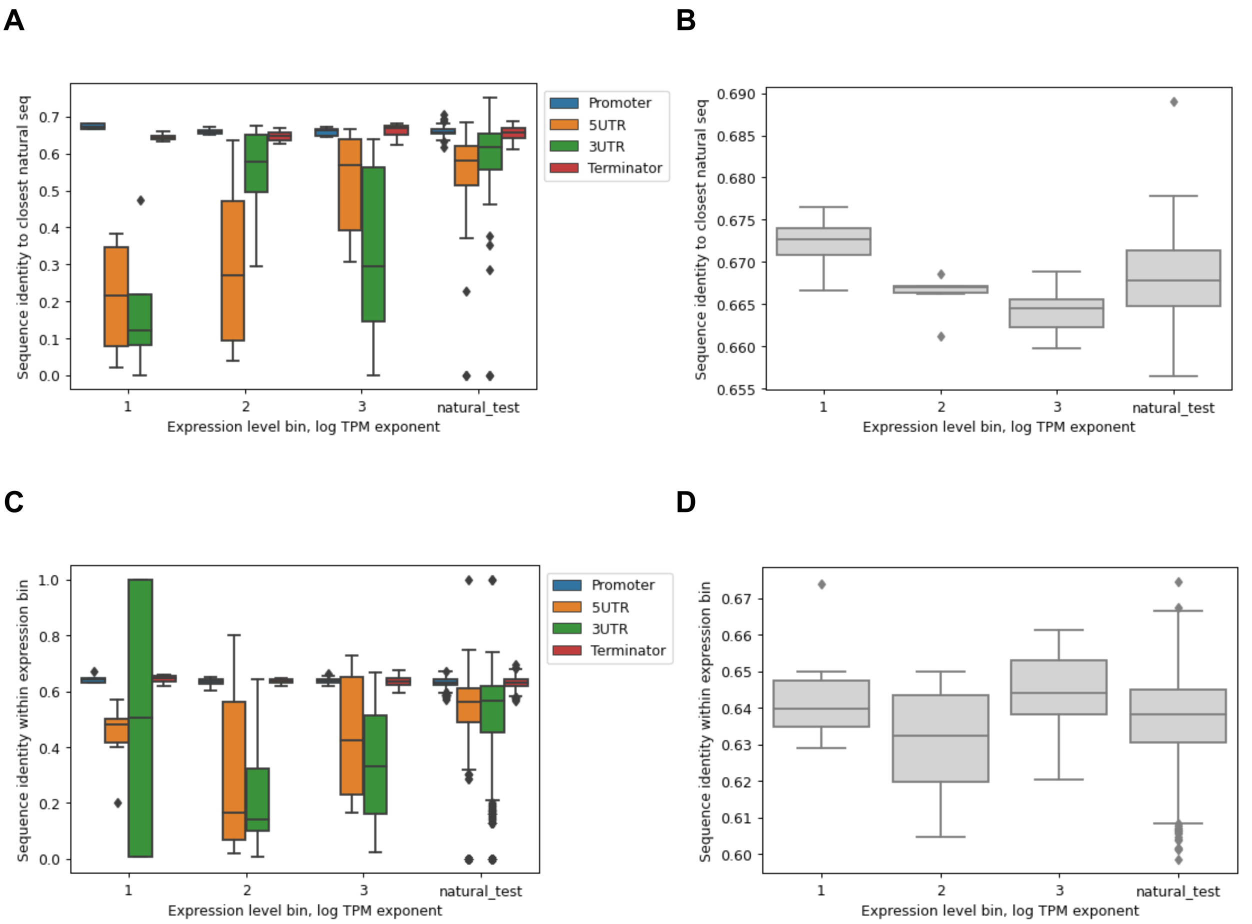

Supplementary Figure 15. Sequence identity of the experimentally validated generated variants (see Supplementary Table 4) and natural test set sequences (A,B) to closest sequence in training dataset (*n* = 16, 24, 28, 256, respectively) and (C,D) within experimental bins (defined by predicted expression levels of generated sequences; *n* = 24, 60, 84, 8064, respectively), computed across (A,C) separate regions of the regulatory structures (promoter marked blue, 5'UTR orange, 3'UTR green, terminator red) or (B,D) whole regulatory sequences. Boxes denote interquartile (IQR) ranges, centres mark medians and whiskers extend to 1.5 IQR from the quartiles.

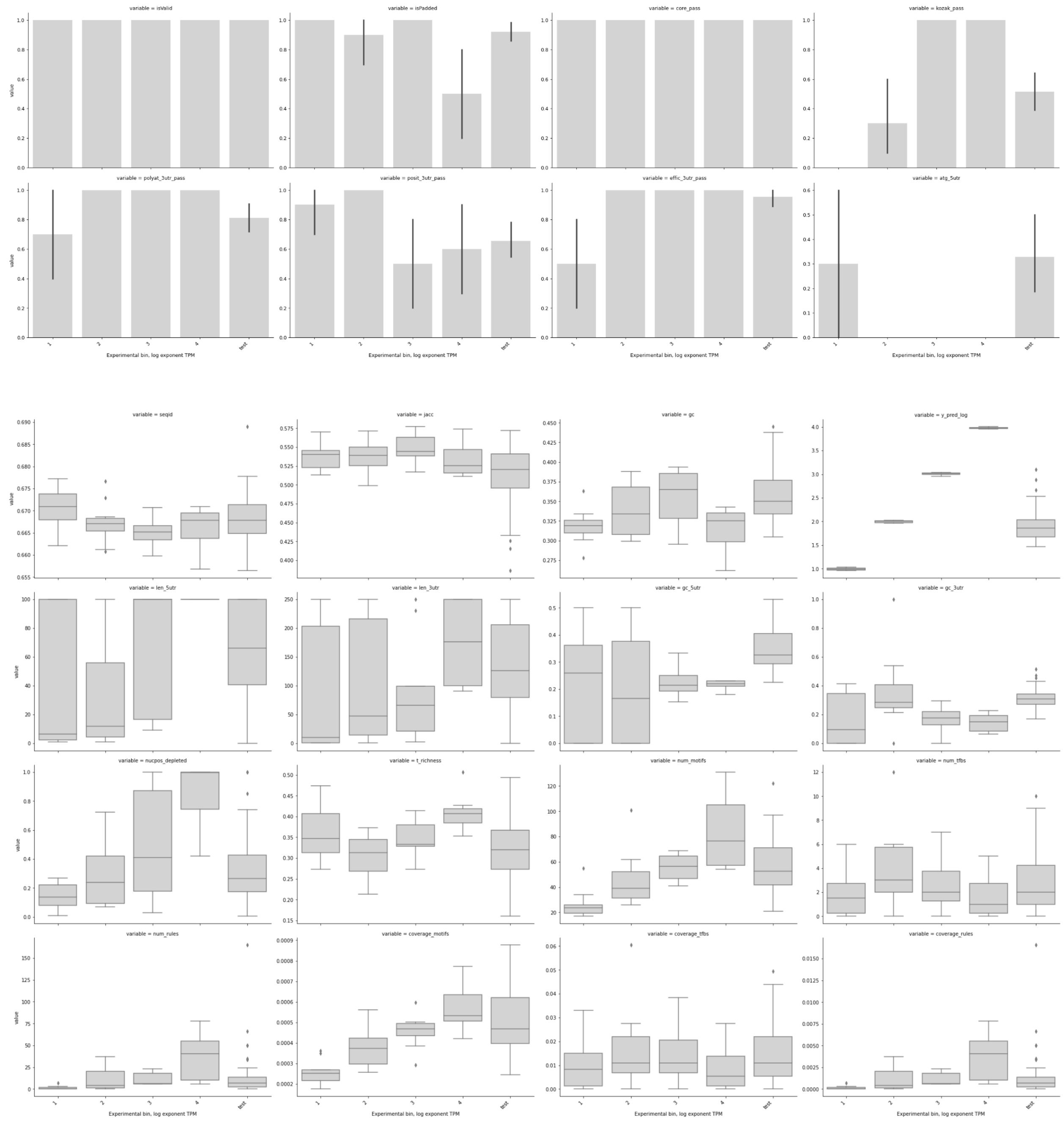

Supplementary Figure 16. Computed sequence properties (see Supplementary Table 3) of generated variants sampled across 4 orders of magnitude of expression levels (see Figure 3C: predicted TPM of 10, 100, 1000, 1000; *n* = 64 each). For bar plots, error bars represent 95% confidence intervals. For box plots, Boxes denote interquartile (IQR) ranges, centres mark medians and whiskers extend to 1.5 IQR from the quartiles.

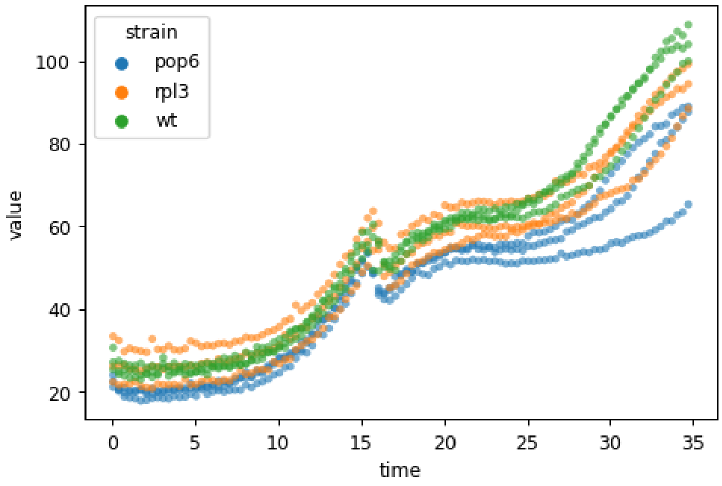

Supplementary Figure 17. Biomass measurements using a bioreactor (Methods M5) showing that the GFP gene did not affect cell growth. Different strains are color-coded: POP6 blue, RPL3 orange and WT green.

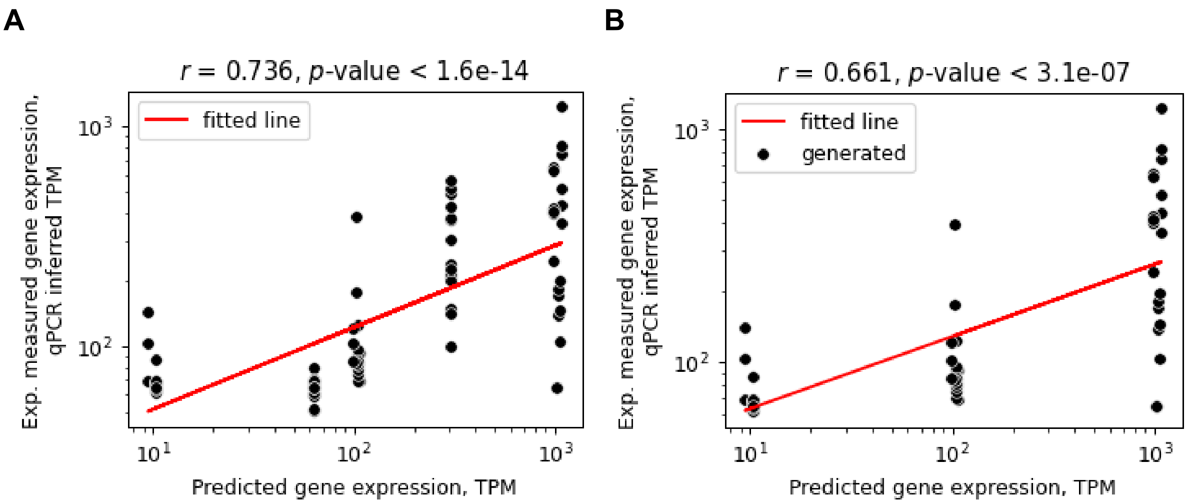

Supplementary Figure 18. Correlation analysis across experimental bins of generated sequence variants (A) including all measured constructs (generated sequences and controls) or (B) including only generated sequences. Red lines denote least squares fit. T-test was used.

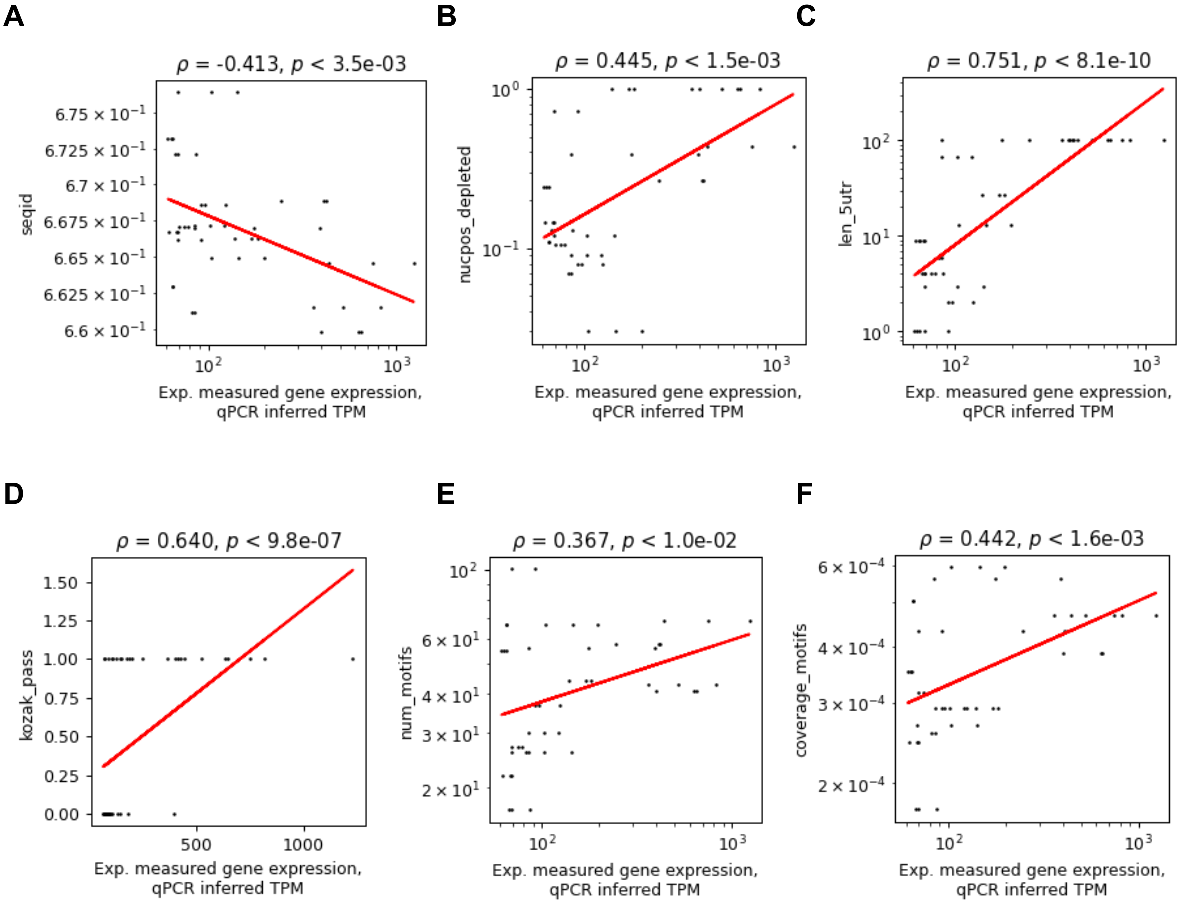

Supplementary Figure 19. Correlation analysis between experimentally measured mRNA levels and predicted DNA sequence properties: (A) sequence homology to the closest sequence in the natural training dataset, (B) proportion of predicted nucleosome depletion [9,10] across the whole sequence, (C) length of the 5' UTR region, (D) presence of a Kozak sequence [4] as well as (E) number and (F) coverage of known expression-related motifs [8]. Red lines denote least squares fit. T-test was used.

A

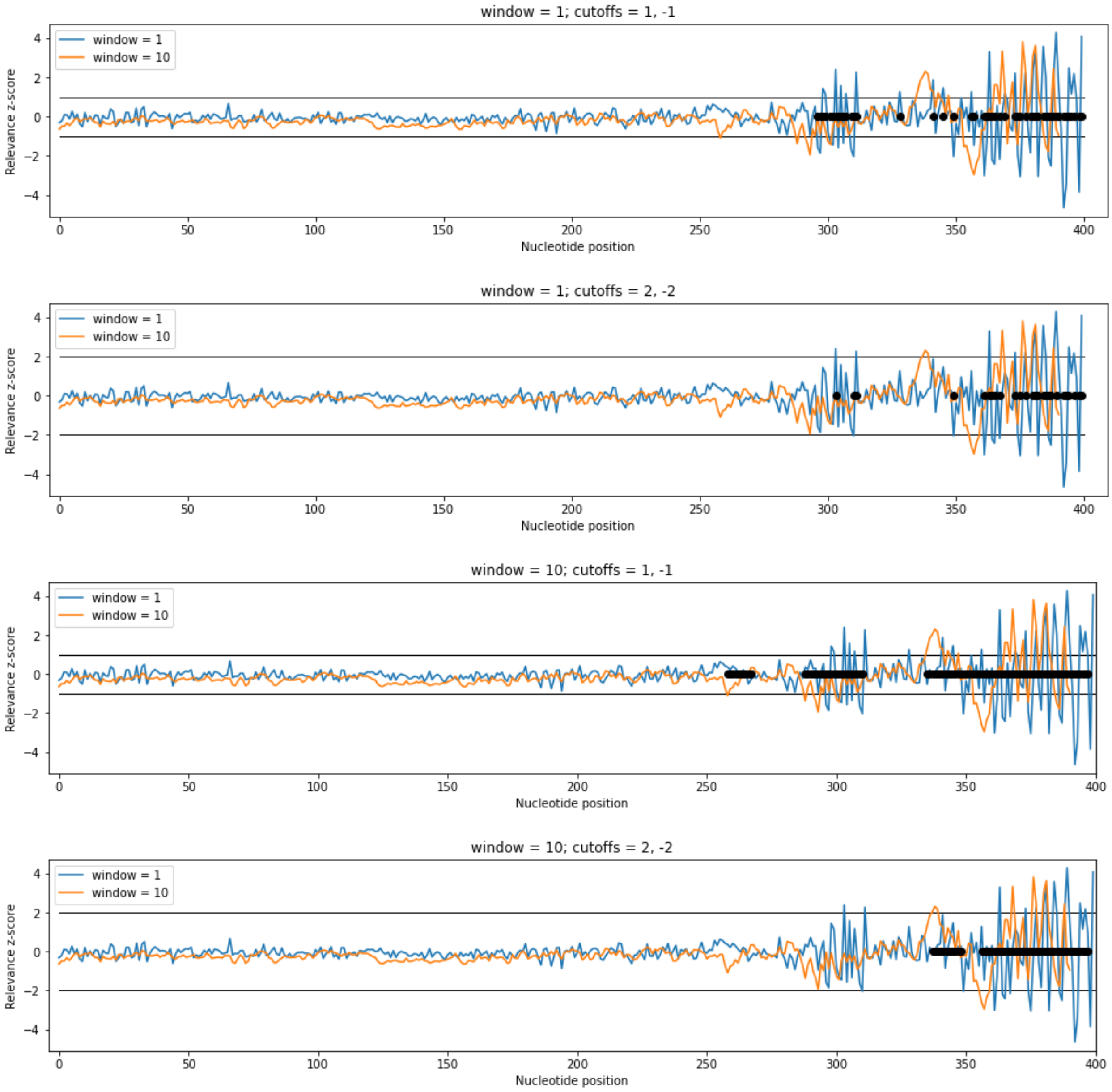
window = 1; cutoffs = 1, -1
window = 1
window = 10
Relevance z-score
Nucleotide position
window = 1; cutoffs = 2, -2
window = 10; cutoffs = 1, -1
window = 10; cutoffs = 2, -2

B

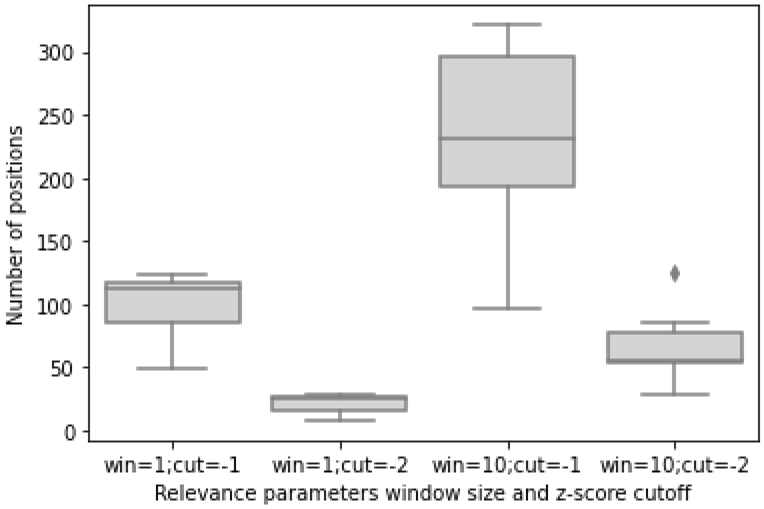

Supplementary Figure 20. (A) Visualization of relevance profiles and relevant positions in the regulatory regions of the RPL3 gene, with window sizes of 1 (blue) and 10 (orange). (B) total number of relevant positions, at different window size and z-score cutoff (*n* = 14 each). Boxes denote interquartile (IQR) ranges, centres mark medians and whiskers extend to 1.5 IQR from the quartiles.

A

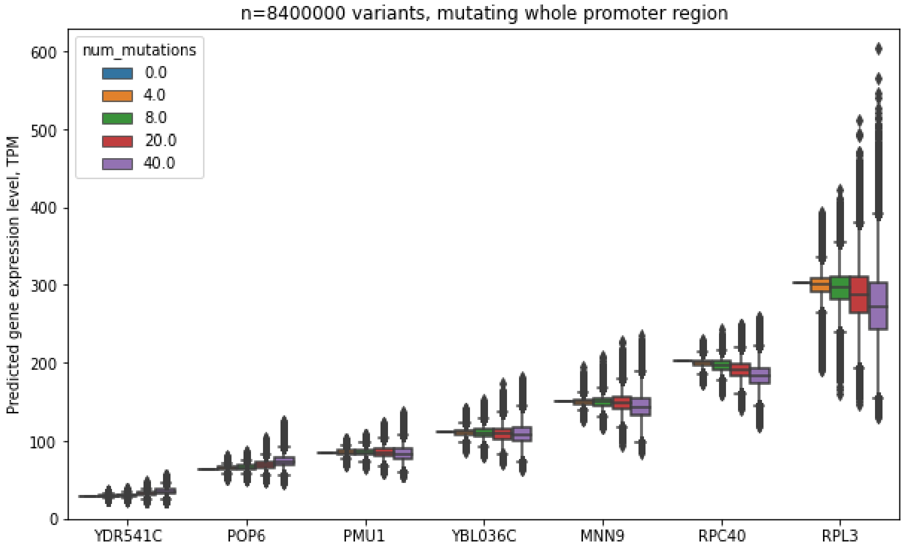

B

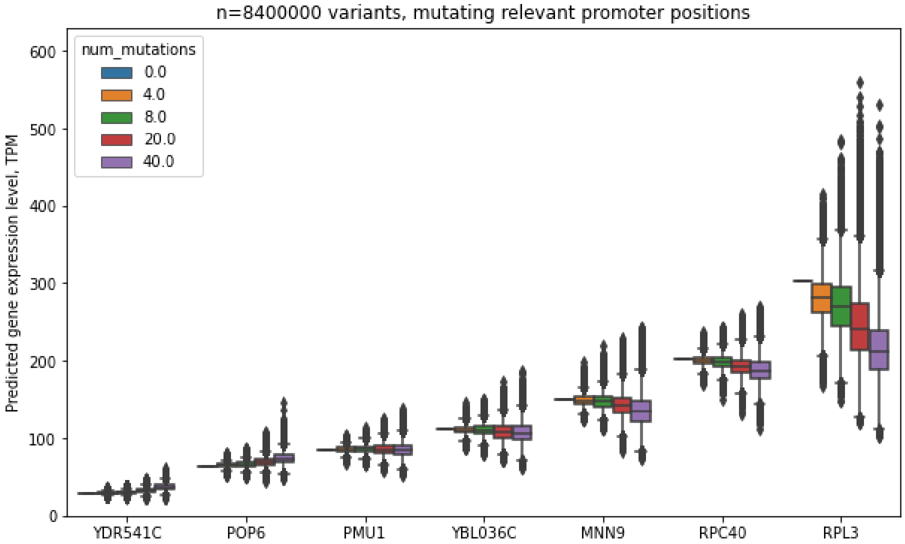

Supplementary Figure 21. Predicted gene expression levels across sequence variants obtained by mutating (A) whole promoter regions and (B) only the most relevant positions as defined by querying the predictor's sensitivity (*n* = 300,000 each except with initial non-mutated sequences where *n* = 1). Apart from non-mutated sequences (blue), the numbers of mutations of 4 bp (orange), 8 bp (green), 20 bp (red) and 40 bp (violet) correspond to 1%, 2%, 5% and 10% of mutated sequence. Boxes denote interquartile (IQR) ranges, centres mark medians and whiskers extend to 1.5 IQR from the quartiles.

A

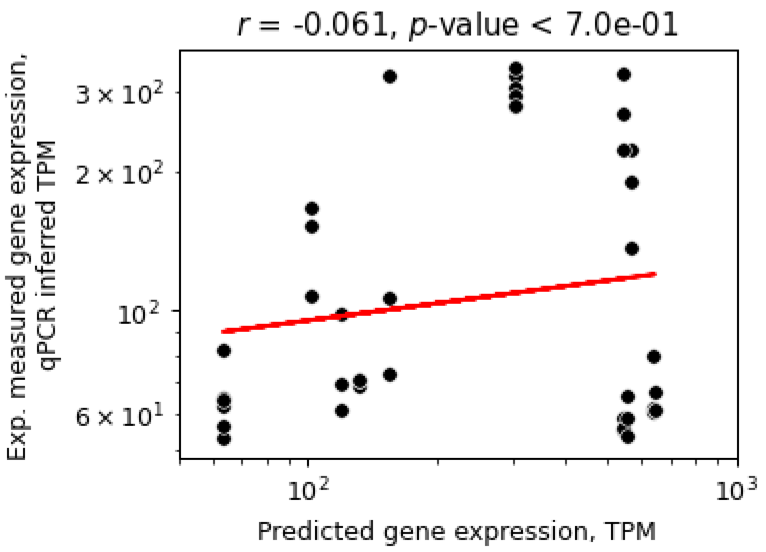

B

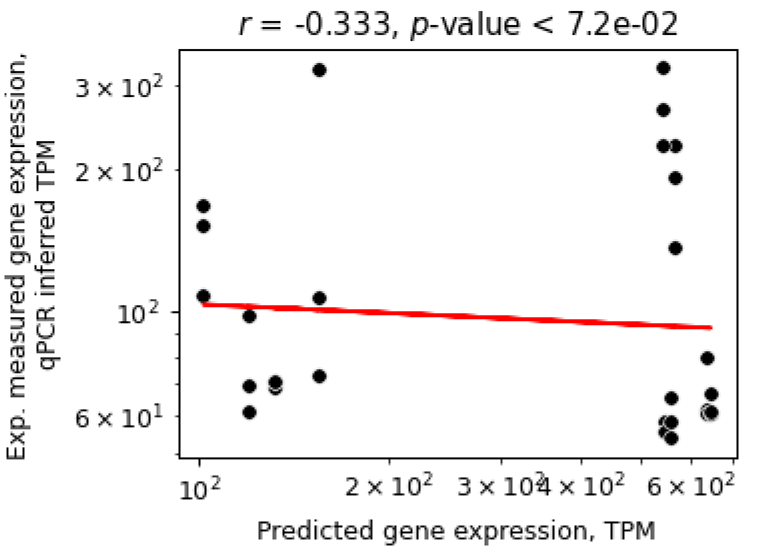

Supplementary Figure 22. Correlation analysis of experimentally tested sequence variants from the mutational approach with (A) included controls and (B) only designed constructs. Red lines denote least squares fit. T-test was used.

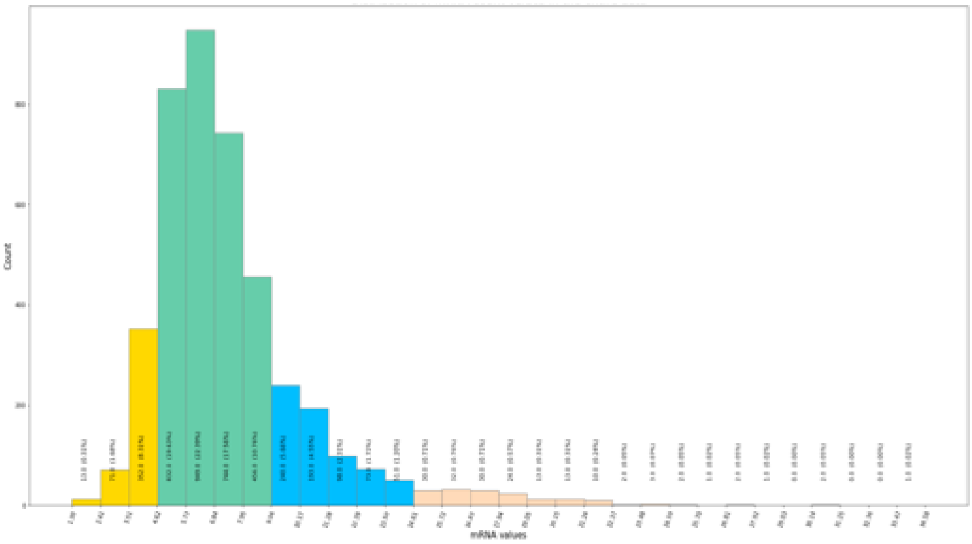

Supplementary Figure 23. Binning the generative model training data across mRNA counts to perform data balancing. Left tail of the distribution (box-cox transformed mRNA level values < 4.62) is marked yellow (4.62 < mRNA values < 8.04), center green (8.04 < mRNA values < 24.35), early right tail blue and late right tail (mRNA values < 24.35) orange.

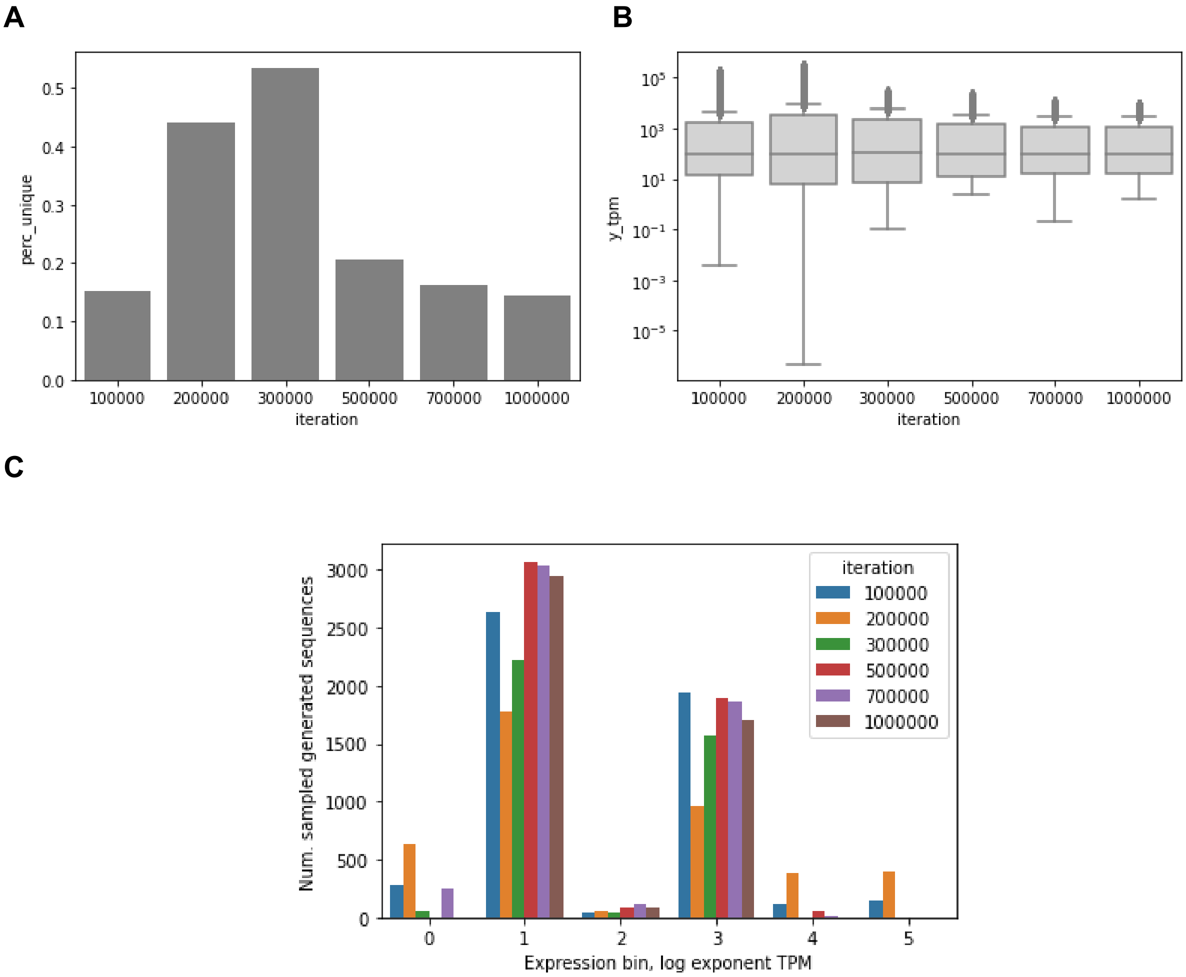

Supplementary Figure 24. Generative model selection by comparing (A) the percentage of unique generated sequences, (B) the range of predicted gene expression levels (*n* = 64,128 each) and (C) the number of generated sequences that could be sampled across a range of 6 orders of magnitude of predicted expression levels per generator. Number of iterations in (C) is color-coded: 100,000 blue, 200,000 orange, 300,000 green, 500,000 red, 700,000 violet and 1,000,000 brown. The generators were obtained at different amounts of training iterations and were further optimized with the predictor-guided optimization procedure for 100,000 iterations (see Methods M4). The generator at 200,000 iterations produced the widest range of expression levels with highest amounts of samples in the more extreme expression bins. For box plots in (B), Boxes denote interquartile (IQR) ranges, centres mark medians and whiskers extend to 1.5 IQR from the quartiles.

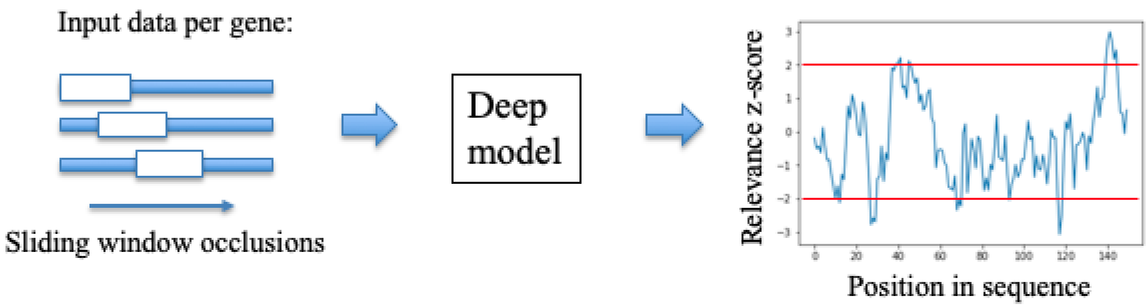

Supplementary Figure 25. Schematic overview of the implemented DNA sequence occlusion-relevance approach [8,23]. Red lines denote z-score of +/- 2.

Supplementary Tables

Supplementary Table 1. Verified DNA sequence properties. Properties with underlined variable names were used in the sequence selection procedure.

Type	Variable name	Description
seq. similarity	seqid	Ratio of Levenshtein 'edit' distance to longer seq. length
seq. similarity	jacc	Jaccard distance
seq. composition	isPadded	Check if sequence contains Ns
seq. composition	isValid	Check if sequence contains Ns in correct positions (UTR borders only)
seq. composition	gc	GC-content
seq. composition	len_5utr	Length of 5' UTR region [15,24]
seq. composition	len_3utr	Length of 3' UTR region [15,24]
seq. composition	gc_5utr	GC-content of 5' UTR region [15,24]
seq. composition	gc_3utr	GC-content of 5' UTR region [15,24]
seq. composition	t_richness	T nucleotide richness in region from TSS to TATA box (region 0 to 150 bp from TSS analyzed) [3]
seq. composition	homopolymer	Presence of homopolymers of length >9 bp, as they can limit synthesis
regulatory grammar	nucpos_depleted	Nucleosome depletion based on R package nuCpos [9,10]
regulatory grammar	core_pass	Presence of core promoter sequence 5'-TATAWAWR-3' [2,3]
regulatory grammar	kozak_pass	Presence of an A-rich Kozak seq. in 5' UTR of 5-15 bp [4,5]
regulatory grammar	polyat_3utr_pass	Presence of poly-A/T seq. in 3' UTR or terminator [7,20,25]
regulatory grammar	posit_3utr_pass	Presence of positioning element 5'-AAWAAA-3 in 3' UTR or terminator [6,7]
regulatory grammar	effic_3utr_pass	Presence of efficiency element 5'-TATDTA-3 in 3 'UTR or terminator [6,16]
regulatory grammar	atg_5utr	Presence of upstream ATGs in 5' UTR [15,26]
regulatory grammar	num_motifs	Overall num. relevant sequence motifs identified from reference study [8]
regulatory grammar	num_tfbs	Overall num. TFBS from the Jaspar and Yeastract databases [1,27]
regulatory grammar	num_rules	Overall num. motif association rules identified from reference study [8]
regulatory grammar	coverage_motifs	Coverage of relevant sequence motifs identified from reference study [8]
regulatory grammar	coverage_tfbs	Coverage of TFBS from the Jaspar and Yeastract databases [1,27]
regulatory grammar	coverage_rules	Coverage of motif association rules identified from reference study [8]
model predictions	y_pred	Predicted gene expression level [8]

Supplementary Table 2. Experimental validation (Methods M6) of designed sequence constructs with the generative approach (Methods M4).

construct	bin	batch	predicted TPM	avg. 2pddct	qPCR inferred TPM
5	gen ~10	5	10.33	12.65	63.12
6	gen ~10	5	10.41	28.35	66.47
7	gen ~10	5	10.35	64.67	74.22
10	gen ~10	4	9.45	13.95	104.65
15	gen ~100	2	104.04	51.69	83.33
17	gen ~100	4	97.94	13.64	102.90
18	gen ~100	2	103.65	36.74	74.23
19	gen ~100	2	103.86	85.74	104.07
20	gen ~100	2	106.48	47.35	80.69
23	gen ~100	4	103.33	33.72	216.87
26	gen ~1000	2	1,027.04	21.78	65.12
27	gen ~1000	3	1,055.05	9.38	149.07
28	gen ~1000	1	1,046.14	9.70	163.82
29	gen ~1000	1	989.01	39.54	558.62
31	gen ~1000	1	1,076.07	58.27	806.45
33	gen ~1000	4	1,077.47	95.58	567.97
34	gen ~1000	3	985.57	27.30	358.57
POP6	ctrl low	1	63.63	2.13	63.63
POP6	ctrl low	2	63.63	19.33	63.63
POP6	ctrl low	3	63.63	2.08	63.63
POP6	ctrl low	4	63.63	6.72	63.63
POP6	ctrl low	5	63.63	15.05	63.63
RPL3	ctrl high	1	303.20	20.24	303.20
RPL3	ctrl high	2	303.20	412.72	303.20
RPL3	ctrl high	3	303.20	22.56	303.20
RPL3	ctrl high	4	303.20	48.93	303.20
RPL3	ctrl high	5	303.20	1,137.72	303.20

Supplementary Table 3. Number of sequence variants with >50% predicted change in gene expression levels obtained using the mutagenesis approach.

region	window size	z-score cutoff	percent mutation	increase	decrease
relevant	10	1	1	0	0
			2	0	0
			5	0.000013	0.00001
			10	0.000087	0.000667
	10	2	1	0	0
			2	0	0
			5	0.00001	0.000003
			10	0.000097	0.000107
	1	1	1	0	0
			2	0	0
			5	0.00004	0.000037
			10	0.00009	0.00158
	1	2	1	0	0
			2	0	0
			5	0	0.00002
			10	0	0.000027
whole	/	/	1	0	0
			2	0	0
			5	0.000007	0
			10	0.000077	0.000517

Supplementary Table 4. Experimental validation (Methods M6) of designed sequence constructs with the mutational approach (Methods M3). Relevant positions were determined at window size 10 bp and z-score cutoff of 1.

construct	bin	strategy	mutatated sequnce size, %	batch	predicted TPM	avg. 2pddct	qPCR inferred TPM
3059399	increase	relevant	5	2	555.58	65.11	59.21
3080857	increase	relevant	5	2	645.09	86.43	62.81
3438152	increase	relevant	10	2	635.45	113.70	67.41
3480767	increase	relevant	10	2	544.09	48.97	56.48
8207535	increase	whole	10	1	541.38	1,689.55	273.57
8248101	increase	whole	10	1	564.84	975.18	183.82
3000527	decrease	relevant	5	2	119.88	165.75	76.20
3529331	decrease	relevant	10	1	101.67	644.39	142.25
7861215	decrease	whole	5	1	154.61	850.87	168.20
8172100	decrease	whole	10	1	131.41	71.11	70.22
POP6	ctrl low	/	/	1	63.63	18.63	63.63
POP6	ctrl low	/	/	2	63.63	91.29	63.63
RPL3	ctrl high	/	/	1	303.20	1,925.34	303.20
RPL3	ctrl high	/	/	2	303.20	1,509.69	303.20

Supplementary Table 5. Hyper-parameters used with deep learning algorithms. CNN denotes convolutional neural networks, FC fully connected neural networks, and LRS learning rate scheduler.

Type	Parameter name	Values	Value range
Global	num epochs	500	fixed
	early stopping min delta	0.01	fixed
	early stopping patience	50	fixed
	LRS epoch drop	10	fixed
	learning rate	(0.00001,0.1)	log variable
	beta_1	(0.5,0.95)	uniform variable
	beta_2	(0.9,0.95)	uniform variable
	epsilon	1.00E-07	fixed
	mbatch	[64,128, 256]	fixed
CNN	kernel size	[10, 20, 30, 40]	fixed
	filters	[32, 64, 128]	fixed
	dilation	[1, 2, 4]	fixed
	stride	1	fixed
	max-pool size	[1, 2, 4]	fixed
	max-pool stride	[1, 2]	fixed
	dropout	(0, 1)	uniform variable
FC	dense size	[32, 64, 128]	fixed
	dropout	(0, 1)	uniform variable

Supplementary Table 6. List of generated sequences from the final set of selected variants, where at least the promoter or terminator could not be synthesized by Twist or IDT at the time of purchase.

construct	sequence
promoter_TPM10_01	AATTCATCGCCCAGTGTTTAATTGACAACATTTGCTTTTTAGAATTTCATGAGCTCTTT<br>AGTCCAGTTTTGTCATCCTTGTGTGCATGATGTATGTGCCAGATTTATAATTGAAGAA<br>AATTTTCCACATAATAATGCGAAAAATTTCGGAATGCTTTTATTGTCGACAGGAAACTC<br>CATATACAAGTGTTTGGGTCTTTGCAGAACGACGAGGGATGTAAAAAATGAGTGGCA<br>TCCATTCAAATGGATTTAAATATTTGAAAGAGTAGAGAAAGACCTTTGAACATAATAAT<br>TCTTTGTTTAAGTTAAGCAATTAGCATTGCTCTACAGTATAGAAAACAGCAATTTTTAAT<br>TAGTTCCTTATATATAATTTATTAATTGCGTTTATGGTCAGAGGTAAAAATGCGCTCCCC<br>TCCTTCATCGTTTTCCGATTTCCTTCGTAAGTGCTCTTTTTTAATAGTATTATTGCAAAA<br>GAGGGGGTTTATTTCGTCGTAACCACAA
terminator_TPM10_01	ACAAGAAAATTTCTAAAGGGTGGTATACAGTAATCTATAGCATATAGGTCTTTGCACAC<br>TTTATTACCCATGGGATGAGATTGCATTTACTGGTAAATGTATACATCTGATTGGAAAT<br>TAGTGAAGATGACTCGATTCCGTTAGTATAAATAAATTGTCATAAAGAGGTGGTATGG<br>GAAATTTTCCTTAGAAATGAAAAGAAATAAAGTTTTTGCTAAAATTGACGAAAATTTCT<br>TTTGCTTCTTTGTACTAGTTTTTTTATACAT
promoter_TPM10_02	CATTTGATACAGATTCCTTAAAACTTTCTTTTACAAAGGCTATCATTCAAATATGGTTCA<br>GGAGTATGATATTTGCCGCTCGCACAGAGGCCGTAGTGGTATATTTGTGATGATATCA<br>AAGCGAAGTACTGTGTTAGAATGTACTGGCAGTATAGATGCTTCTAGTGGAGTGAAC<br>TTTATATTTTCTTTTATTTTTCTTACTGAGGTATTCTCTTCTTATATCTATGAGAATAAGA<br>GACCACCCTCAGCACTAATTTAATAAGTATGTAGTAATGTTTACATTCTGAAGTTCACA<br>TTAACTATAAAAAAATAGAAATAATTCCTTGGCGTTTGCAACTTTTATAATATAATAAAGT<br>TCAACGATCAGCAATAAAAATAAAAATTTATGATTGATGGCGTGGGGAAGGTGAAAAT<br>TATTTTGAGACTATTATTGAAAAAATGAGTTTTTTCGTTTTACAAAGAAATTAGTCAAAT<br>TAAATAGTTTTTTCATCATTCTTTCA
terminator_TPM10_02	TTTGAACTTGTTTTACCAATGTTGTTTGGCCCTTCTTTGGAACTTCTCTGATACCGGG<br>GTAGGTTACCGTACAAACGAATCAGAAGAAAAGACATTATAATGTATATTCCATTCCAT<br>GATCAAAGGGACGAGCAGAGAATCTACCTGTATAATTAGTATAGTGGGAAACATCTC<br>CTTAACTCTAGGATTAAAAAGGGGGAGCAAAAAAAAGAAACATGAAAATAAGCAAATT<br>AACCGTATCAATTAATTTAT
promoter_TPM10_03	ACCTTACGCATCTATATGTGCTTACGAAAAGATAATTTTCACAAGAACTATCTTATATAT<br>ATTCTCTCTCCAAAAACAAAACACTCAAAACCGTTAGATCTATCAATGTGAGCCAATG<br>GATTTTTCCTCCAACGCAAGAAATACCCACCGACAAGATTTATAGTGTCCTAACCCAT<br>TGCAAAAATTGGCTCTGCTTGGGCCATGTTGACGAATTTAAATTTAATTGTCAAAGAA<br>TAAAGAATTCAATCAGTTTTAAGAAAGGACGTCTCACCCAGGAACTATAAATAAAATTT<br>ATAACATATTAATTGTGTTTTAACTTTGCTATGTCTTTCTTTTTCAATTGGAAAGAAATC<br>TATTTTCTCACCCTCTTCTAATTTTTATTAGTAAAACCAGTTTATTCTAATTCGTGTTACT<br>TTAAGAATCCTTGTCTAATATAGGCAATAAAATAAAAACACACCACCAAACAGGAGAC<br>CGATTGTCCAATGAAGGGACCGAAGCTA
terminator_TPM10_03	TATGTTGTTTTGTTTGTCTCCATATAATAAAACCGCCATAAGGCATTCTTCAGAAAGTT<br>CTTTGAAAAGTGGCATCTTGACCAATTTCCCTCAAAACATGCAATTATGATTATCCCG<br>TACGCATCCCACCCTCTCCCACGACCAGCCACTCTAACCATGTTTTCATAAGTCGGC<br>TGATTTTGACATGTGTATAATTGCCTTTTTATTATGATAATCTTGAAATTACCATGTTATA<br>TGGAATTATACCATTCTCAATAATAAATTAGTTCGTGATTTTAGAAGCCTGGTAAGCAA<br>ATGATTAATTCTCTGAAC

promoter_TPM10_04	TGTAGAAACTCACAGCAACAATTTCAGGGACAAAAAAATTAAGGTAATCTTGATCCAAAAACTCCAATGGAAGTATCGGAACTTTAAAGGGCCATGAGAGCAAAATTGAACAAACAATGTCTGCGAAAATTTCAATCAAAACTTCTTTGTATTAGATTGTCATTATATTTATTTTGAAGTAAGAAAAAAATATATAACTGTATTAGAACTTTATTGACTGTTTCTCTCTTTTTTGTCGGACCATACCTTTAGTTTTCTATCGTTTTTTTCTTTTTGCATCTTATTTCTTAGCCTGTCGTTCTACTTCATTTATTTCTTTATCTATAAAAGTCTTTTTGCACGAGAACCATATTATAAGGTACTATTCAGTAAAATTGATACTTATAAATGCCGAACTTTTA
terminator_TPM10_04	AGTGGTACTCTAGCATGCGTACCATTTCTTCGATTATATCTTTTTCTTTCTGGAGATGCGCTTATTCGGTCGTCTGTCACATTGAAACAAGGATGTCACGCTAAAAAGAAACACTGTTATACTGATATGGAAAGGACACGTTTGCTATTATTTTGTCATTCTTTATGCTTTATTTGATAGCAGAATGAGGAAAATATTCCAATTGTGGATATATAAGTGGAACAGAGATGACCATAGTGGATAAAAGAAAATCGAAGAATTTGTTTAAAAAAAGCAGAGTTTGGAAAGGGGAAGTTGATCTGCTAGCAGCATTTCACAATGAAAAAAAGGCTTCTGCACGAAGAATTGTGTGAGAAAAAAATAATATCAATCTTCCATACAATTTCAGTAATACCCAATATACAGAATTTTGGAGGAATGAATAAATTTTGTTTATATCAAAAATTTTATTCATCGCTTAATACACCTCAATTCAATTACAATCAGGAACATCTATCTTA
promoter_TPM10_08	ATTTCACTTAAAACAGAACACCTATAATTGAAATGATGTTGTTCGACGACGCGTGCGCGCTCCTTATACCGGAGTAGCTGTTTTTGTATTTTATAACTGTAGAAGTTATGTGAACTTATATATAGCTTGCCTCCAAAATTCTCTAGATGTTGATATATCGGAACTTGATCCAGATGTGAATCTACAACTAAACGGTTAGATTTGGAGTGGATTCCAAAAAGAAGAACGGTGGCATACAAATTCGAATATATATTTAGGATTAGACTTATACGTATGCAACTCTATTTTGTTGTATCACACGCGATCAATAGAGAAAAGAGCACCGCTATCCCCACTCCAAAGTAAACGACACTACACATCAGATCAAATAAGATATTTAAAAACAATGGAATCTTTAGATTTTCATTTTAGTGTATTAGAAAGACTTTGCGGACAAATAGAAAGGAAAAGCAAACACAAAAAAGAAAAAAAACAAGAAGTTAATCTTTGTCAACGCCAAA
terminator_TPM10_08	TTTTTTGTGCAAATGATAAGTATAAAGCAGTGGGGGCTTCATGGACGGTTTTGTGTTGAAAGAAGGGGAGGTATTTCTGCTACTTTCTAATTTTTCTGACCATTTGATAGCATAGAACACCTCCATCACACTAACATGTATCAAAAAAAAGAAATTATATCAGATATCAAAGGAGTTTGAAATGATTTAACAATTTACTCCTTGTAATCTTTGTATTCATTTAAATTATTTAATTTTTTCTAGCTATAAAAACA
promoter_TPM10_09	ACTTTGAGAACACTCAAACTGACGATTGAAAATGTAAGCCTTCACGTCATCACGATCAGCGAAAGGAGCTGTTGTTATGAACATTTTAAACACAGATGATATAAATAATAAGAACTTAAAAAGTTTTTATTATTTCTTGTTTACTATATTTTTTAGCATTTAATTGTAAGTAACAGGGAATTTAATAAACTGGATTGATGGTTTATATACTACTTTTTATATTTACTAGAACTGACGATAGTTGAAGTCGTATAAACAATACTTTTCCCTTTTCTCTCAATTCTTTGCTTAATATAGACCACTCGAATTCCTGAATTAAATTTTTACAACTTTCTGCTGTTAGATCAAAACGAAAATAAAAATAAAATAAAAATGTTCAAAACAGCGTTTCAGTAAAAATTA
terminator_TPM10_09	TAACCTTTGTCTCTCGGCAGCATTCGGCAAGATCGGGGAAGGTAGAAGAAACTACGAACTCACAATAAAATTCTCCTAGAAGAAAAACGCAGAAATACATGCGAAATTATTACTGCAACCCTTCGGCATTTACCCTGAAACTTCATACCCCATCATTGCCGCATCCTAACCAGTTCATATAAAGTAGAAAATGACAAATGACACCTTTCAATTGCAGGTCATGATAGAAGACTGTGGGCGGACATTACTAGCCTGTTGACTTCTTTTCTCTGTAAAGAAGTCTTTAAGGAAGGATTCCATACTTGCACTTCTTTCTCCAGTAAAATTACTCATTCGAAATTCATGATAACCAACGGTCTCAATTCATGGTGCCTTTTAGGAAAGGAGGAGTATGAAGAGACCCAATCTTCAGCTGAACACGTTGAAAACTTAACAGAATTTAAGTAAGGAAAAGAATCAAAAAATACATATTTAATTAAATTTGTTAATAATACATAACC
promoter_TPM100_02	AAGCTTATTATTATACGGAACAAATTTCCTTATCGAGAAATTCTTTTGCGGAAGATTAC

	AGGAATTTGATGTTGTATATATTGAATAGTACAATCAAGATCTTGATCTATTTAAATATG CATACGCATCGAAGCAATTTTGATGAAGTTATTCCTAATTAGTAATTGTTGCTATAAAC CGTCGAACGACTAGCCGAGAGGAGCTCAGACCACAGGCTGTTAATTCTAAGGGAAA ACTAGATATTACACTAAAAACGCCTCGACTATAAAACTTGACATATTTTTTGATTTTTTC GTCTGGTCTATGACTTTCTCGATTGCTTTTTTTCCCCCAAGAGATTAAGGTGAAAGG AGATTTCAAATACGATAACCATTAAGAAATTAGTTGAACAATACTACTAAGAGTACCAG ACAACTA
terminator_TPM100_02	AATCTGTGATATTTATTTTTGTACCAATATTAGGTGATTCCTATTTTTCTGATTTTTTTAT ATAAATAACTAAAAAAGAAGTTGCAAAAATAAAAAAAACTGACAGGTAAATGCTGCCA GGATAAATAATCTAAAAAAAACTTTCCTAAGAAGTGAAGAAGATGTAATGTATCCTGAA GTAAGGCGATATAGAGGAGAAGGAGAAAAAAATCATAACTCATAAAAGATGGCTAAAA AGTTGCGGAACACCT
promoter_TPM100_07	CTCAACTCTGTTTAAAGTTCTCTCTCAATCGCTAAAAAAAAACTTTCGAGTAGTGATG GCAGTGGCGTAAAGAAGAGATGGACCGGCCATAAGCACTAAAGACCAATCCAGACG GTCACACCAGAAACGAGAAAGCTGAAATTACCCGACTCTACTATACAAATCAGCTTT CATTTACAAATAAAGGTAGCAATGGTAAACCATGAAATCACGCGGTAGCTACGGATAT CCATTACACCGCTATCCAATATGATCTGTAGAAAGGAACTAATTTTTTTCGCTTTTACT AATAGTACTCGCTATTACTACTTACCTCTGCTTTTTTTTCTAGTCAGAGCCAATGTGGT ATTCAAAGTTATTGCAGTGCATTTGCGTGGACTGCAGCAAAAGTACCTTACAAAAAA AGGAAGGAGAACTGTTGTGAAAGGGGCATCATACTTGAGTAACCAAAAAACTGGGT ACTTGAAACGCACTATACTGGAAACGAGATAA
terminator_TPM100_07	GGTTAGACTGATTAGATATTATGCTATATTTTCATGTTGGCTGACTTGACACTTATTTAT ACTATTACATAATATATTACTACTTTGCCTAAAATATTTGTTTATGTGGGGTAAGAAACG CTCGTAAATTTTTCTATGAATCATCTGAGCGAAACCTTTAACTTGCGCTTATTAGGAGA AAAAAAACGAATTATGGATAGAGTGGTATGGCAAGCCGTTTCGATGACCCAAATGCG TCTAAACGGTGGCCTATAGTATGAGCATTCGAAGGATTACAAAATGCGGCACCAATT GTTTGAAGGATGCACGGAGTGTGAAAATAAATGATAATGTCCAAAAATAAAAGATATG TATTACAATGCA
promoter_TPM100_08	TTTTGCAACCAAGCCTAAGCAAACGAAAGAGCTCAGAGTTAATGAGCACTTTTTCTA ATTTAAAGCCTAGTAGTGTGTACTTCGCTCAAGTAATCGTTTTACTTTTAGTGTACACA TATAGGTTAGTTGCTAGCACTAGCTGAAATCAAATAAGGTGTTAGGTACGGGACATAT AGTTGAATCAGCTCAACTCTCACGTCTTCAACACCGAACCACGGCCCGAAGTCATG GCACCTCATTTATCACCTTTTGACCGTCACACTTCAAAACATACAGCGGTTGGGCAA TAACAGAAAACCCCCTTACATTCTCTTGTTTTCCTTATTATCACCACCCAAGATTTTCA TCCAACTCAAGATCAATTCAAGAGTTTGTGATGACCGACAAACTAAAGAAGTGAACA ATTA
terminator_TPM100_08	AGAAGAATAAAAGTGAAAGTTTTTGGAGCTTTGTTGGTATTTTCTTCTTGAGAAAAGG GTGTGCCTATGTAGCTGAAAATTATGGGTAGGACCATAGGGAATTTACCTGATGCTTC AAGTGCAATCTCGCCTATGACACATGAGCACTTAACCTGAGGCAAGATACACATCAA TAATAGGCTTCTGACTTATCTTTCGATTATCAATCCATCGTCACATTCTAGCGCAAAGT TTCAAAAAATTAAATATAGTGCTATGTGCGATGGTATCAATAT
promoter_TPM100_10	TATAGTTTTAAGCTGATTGGATACCAAAAAACTACCATTTTATTAATGCCATGGAATTTG AACGAATCATTTCGATAATAGTAGGCGTGAAGGATACGTCCCCAGTGAAACGAAGGA CAGAATGAGGATCAAATTTTCACAAAGGTGAAAAACAATAAGTTTAAAATTTACAATAG CTAAACATGATTTTGCTTTAGTCCAAAAGTCTAGTGTTGGCTCTATTAGTAATGATTTA TTGTTTTTAATAACGCCTACTTCTAAATGTCTGACCAATATTCCTTAGAGAAAAAAAAA TTGAGTAAAAGGAGAGAATTAGAAAAAGAAGGGAACGTATGACTTATTTGATGAAATG

	ATGTAAATGAATTGTCCATTTTTACACATAAATAAACTGAGCAGCCAAATCGTAGCATCGCAAACCACCA
terminator_TPM100_10	AAAAACTGAGATTATACTATGTAAGTTGATGCATATAAAATAGTAATAAGTATCATTTTTTATGTTGACTTTGATATATCATTTTCGCCTTTGGCTAGAAGCGCCAACAAATTTATAAAGGAATTTTCGTATGAATTACTTACCTATATTTAAGTTTTGTCGTTAAATGAATTCATCGAAAAAATATAACGATATACTTTGTGATAACAATTGCCTTGTCTTGCCAATGGAACTTAAATTAACACGTGAAGAGTTTAGTAGTTAGTTGATTGGTAAATAGAATAAAAGGGATACAGATTGTCACGTAAACAAAAAGATAT
promoter_TPM1000_01	AGAAAAAAAAGGTGGGGAGCGGCTGGAAGTGCACTTATCCACCCATATGTATTTGGTATACCTTACTTTTTGTACCATATTTCTGCCTCACCTAAGAAAAACCGATTCAACAATCCGCATATTGCGCAGTTTTAGGGGTAATTGCAATTGAATCTTGCGAACAAAGCCCACCATACCGTTCATCAACTTCCATCCGACCGTAATTCATAAACGCATGATTCATCGTACATTTTATCTTTGCAGAACAAGTGATTCCTTTTCTGCAATCCGTCCTTGTTGTCATCATTCCCAGCCGGTTTCCTACCCCGGCTTCCCTCAAGAAAGTAATAAATTGGAAAAAATATAAAGAGATAACGAAATAGGGTAATGACTTTTTTGTTCTTAGCTTGATTAAATTTACAAATCAACAAAAA
terminator_TPM1000_01	TAATATAATTTTTTTTCATAAGGGAATAATTATTTGAAAAAATTCAAATCGATGAAAAACATGATAGTAACATAGGAGAAACATATATGTAAAGGTAAATTAGATTTTTTATATTACGTTAGAGTTTTTCCACCTCTCCATATAAATTCTCTTTATATATGTATTCAGATTTATTATAGTTCGTCAAATCAAGAACAACAAACAATCGTTCAATGTATATAAATTTAGTGCATATATAAGATAAAGAAATTGAAACCTATTTATAGAGGGCTGTGTTTTTCACTTTCTTAAGGCAGGCCTTTATTTATTGTATTTCTATATATACATAGGGTACTGCTTGTTGGCATGCACTACCTGTCGCAAAATGGAAGAGATAAAAACGATCCGCAGAATTTCACTTTCAGATATTAACTTGTCGGGTACAACTACTTTGACTAGCATCTAAAAACTTTTTATTTATTCATGTACTTCGTCCTGTAGAACAGCAACTCCCTTGGCCG
promoter_TPM1000_06	ACTAATTTTCAGCTATACCGTGAATGTCTGGTCTTAAGCACCATTTAAATCGAACATACCGAACACGAAAAATAAGAATTTTTAAAAGTGAGTCAACACAAATAACTGAGCTAAATTCATCACCCTCCTATTTCTCCAAGTGAACTGTGAAAGTTGCACACGGAAAGCCCTTGCTACAATTCCATTGTTCTTTACCCGACTGCTAAACTTTGTATAAAAATAATGCATCCCATCATTCAACTCCTTCTCTTGCAATTTCCTCCCGGCATTTACTCGCACTTTTCACTACAGGTGGGGAATGGGCCACGTTTCCTCACCCCAAAGAAAGGAAAAAAGAACGATATATAAAACTTGATAATAATGTTGTTCTAAGTTTTACTAATTTTTTGTATGTTCTAATTTACCAATAAAGCAAAAGTAGCATTAAAGCATTATATTAATTAATTAGGTAGATTAAGATTGGAATACATTAATTAACTGAACAATAACATAACAAAACA
terminator_TPM1000_06	ATTTATAGAATAAATATTTATAATAACTTAGTTTGATTTAGTTTAAGCAATTATAGTTTATTTTTTTTAATATATTAATCTATTATTATAAATATTATTTTCTAATATAGGAAAAAAATCTTAATAGTTATACTGGTATAACTTATGTTAGCACGGGTTGAAAAAAAAAGAGTAGTTTTTTTGTTTATTCTTGAATTTTACACTATCAGTAGTCTGAATATCGGTGCAGTCTTCAGACAACAACTACAACGACCCACAATTTGTATGAAATTTTTTATTATATGGCCTATTGGCAGCTCATCAAAATACTCCTTTTCGTGCTATCATAAACCTATTAAACATCATAATG
promoter_TPM1000_08	GCCCATACTCATCACTACTTTAATCCCCCAACCACCATCTGAAGGTCCAGACAACCTCGTTTAAACACAGAATTGATACCATAGCTGCATGGACAATCACGAAAAAAAATCACCTCGAATTCCAAAGTGGTCGCCCTTGAGGCAAAACAGGAGAAACAAACGAAAAAATTTAAATAAAAAAGTTATATAGATACTACCCCCTAAAAACTTACCCTCTTATCATAATGAATACTAGGCTTGATAGTCCTCCCTATCCCTCCCCCATTATCCGCCCTTTTGCACGGCGGGGGTCATTTCCCACCTTTCTGTGTCGTAACATTTTTTTTTCAAAGCAAGGTGAAAAAGTGTCTTTCCATAATTTTTTTGATATTTTCTTAACTAGTTGTTTTTCAAAGTACCACTATCC

	AGTAAGATATTTCTCTATTACCATTGAAAGAGTTTAATTTTTAAGAATTGTAATAATTATA<br>AATACACCAAACCATAAAAAACACTAAAAAAAA
terminator_TPM1000_08	AGTTTTAATTTGTCTTTATAATAATTTGTCTTTCAAGTCTATATATATTTTACTTTTTTTTT<br>ATCTTATTTTCTAAAATTAATGATTTTGTCTATGTAAAATATTAGGTATATATATTTTTTTTA<br>CACATTTTTCAAGTCTTTTCTATATAAGTATTAATTATAAGTAACATTCCATCTTTTCGTT<br>TTTTCTTATATTTGTCGTTTTTTTTTCCCTAAATTTATTTTCATTTATCGGCCAATACCCC<br>AAGGAGCTCCTTTGCCCACACCATTTTCATCCAGGAAAAGTTATACTACGCTGAGAT<br>ATCTTTTAAGACTTTTTGGTCATGCCTCTTATGTGCAATCATGAA
promoter_TPM10000_01	TATTGATACTGGCGGGCGGCGCTTCAGTAATACACCCCACGACAGCGCTACCATGTA<br>GAAGAATACAATCAGGTGTGTGTTGCCTAAATGCTACTCGAATAACAAAATCCATTTA<br>TATCTTTCATTTTATTATATTGTATCTAGTTCTCACGTAAACAGAATTTGCGTAAAACTAT<br>ACTACAAAAACACAAGAAAACAAGAATAGATTTTCTTTCTAACCGTTCACCCGCTCAA<br>TAAGAAATATTCCAATATTCCCCACGAAACGTCCCCCCCACCATCACCCCTCCTCTTT<br>TTGTAATATGTTGTTATATTATTTAAGTTGTTTAAGTATCTTTCTTTCCATTTAATTTAGTG<br>GATTATTTTTTCATTTGTTACATTTCTTTTGTTCTTTTGATTTTTTCAACTCAGCTTAACT<br>TATTTTCCTTTTTTTATATTGTTTTTTCTTGTTTTTATTTTTTTTACAAAACAACCCTTTAC<br>ACTCAACTATAATAACAAACAAA
terminator_TPM10000_01	AACCAAACATACATTCAAGAAAAAACATATTAAATTTTTTCTCCCCTCTATCATTTTCTC<br>CTTTCTTAGAAATGGTTAGTAATGTATTACTTCTTAGAAGGAGTTTCTTCCATTTGTTC<br>CTTCTTTTTCAATTTTATTTCAAAAGAGTAGTTTTTTATTTATTTAATAAATAATTTTAATT<br>AAATATTTATAAAATCGTCAAGAACTTAGATAAAATCATTAAGAAGTCATTTACACATAT<br>GGAAAGAGAAATTCTTAAGGTTTTATGGTAATATTTTTGAATAAATAGTTTAATAGGTTA<br>GCCCAATGGAGAGTAGTGGGCTTCCGTTGGAATTGAACAAATTGGTACTCCTTAGTG<br>TTTGTTTCACTTATTTTGCATTTTATCTGCAATTTGCACACTGATGTTTTTTGCAATCAA<br>AAAAGAACTAATGGGCTATCAGCGATCAACTAATAATAATCATTACTGTGAAGGTGCT<br>CCTTTTTGGTACCCCATCTTGGTT
promoter_TPM10000_02	AGAAAAGGAAGACGCCATTTTTGAGCCTGCGATATTCCAAATTTCTTCTACCCATTTC<br>ATAATCCGCTTAAATGAAATTCAGTCATAAAGCCGCAGGAACACACAGAGGCGCCTA<br>CCCCTAACACGTCATCTTATCCATGCCTGTATTCGACCTCCTCAGGCAACTATTTTGA<br>CAATCCCCAAACAATCCAAACACCCAAAAGTAAATCGACCATTGCACTTTTCCAACAT<br>TCACGCCGAAATTTTCCACCCTTCCCCCCTTCCTTCTTGCCTCCTCCTACCTTGCGG<br>GACACCCCGCCCCTGCCCCCCTTGCCATCCCTCATTCCCTCAATTTAATTAAATATTG<br>ATAAATTTTTGTTCTCATTTTCTATTGTGTTTTCTTTTCTTTTTTTTATAAATACTTTTCC<br>CATTAAGATATTCTTCTATTATAAAGAAAAAGTTTTAGTTCGTTAATTATTTTTTTCAACT<br>AAGTATCAAATAACCATACGAACAAAAAACAA
terminator_TPM10000_02	AGTTAATTTATTTATTATATCTTTTTTTTATTAACTATTTTTATCTTATTTCGAATATGTATAT<br>TTTAAATAAAGATTACTTTTTATTAATTTGAAGTGAAGTGAAGGTATAAGTTATGATTCT<br>TATTTCCAAGGTGTCTTCTTTGCCTGAATCAATTAAAAGCAAAGGAATTTTTTGCATAC<br>TAGAAGAAATTGTTTGCCGATACCAGCGATGCGGATGATCCGCAAACTCTTTTGTTA<br>ATCTTTCAATTATGGTAATCAGTAATTTATACATATGAGGGTGTTACGTGGGAGAAACT<br>TTATTATTTGTTTTTTTGGGTTTCTAGCTAATAGAATGTATTTCTTTGAAG
promoter_TPM10000_03	TTTCATTCCGGAAGACGCGTTGAACAGTAACTTGCTCATCCAAACACCCCGCATTTC<br>CGTTCCTCCAATCAAGCCCCCGTATGGTAATAACAGAAATTAAAAGAAACTGATCGTT<br>TAAGAATACACACATATTCGGCAAAAATAAGCCCAATGACTAATCTTAGCCTTACACCA<br>AAAGACCTTATAGTATAGTTTAAAAATTTTAACCCTCCAAACTTATTCCTCAATTTTGCA<br>CTTCTTTTGTTTTTTTCGTCTGCTCATACACTAGGCCCCCCGCCACCATCAGGCTCC<br>CACACGCAGGAATTTCAGTACCTCCCGTTGTATTCGATAAAAAATACGAACTTATTGA

	AAGAGGAAAATTTTTTTTGTTTCTTTCATTTTTTCCTTCTTTTTTTTACACCTTTCTCCC<br>AATTACTTATCTTTTCTTTTTATTATAGGTAGTATATTTAAAGCAATTATTTTAGGAACTAT<br>CTAAGAACATACAATCAAATAATCAAACA
terminator_TPM10000_03	TTCCCAACCACAGATCGTATCCACACACATTTTTATTTTTTTCATATTAAGCAAGTTTC<br>ATCAATATTTTCAACTCTTATACTTAACTAGTTATTATTTAAGTAATACGTAGTCCCATTT<br>TCAAGTTTGAAAAGAATTAAATCAATTTAATTAATAGTATATATAAGTTACTTTATTTCTAT<br>TTTTAAAGATTATTATATCTTTAATTTTCTTAGTATTATCTATTTAACTTTTGTAACAATGT<br>GGTAATTGTGGTAAAGAGTTTTTATACTGATGTTGCCTTGTTGTTGTGTTTTCATTGTG<br>AGTTATAGTTGTTGTCTTATTTCTTTTATTTTTTTCTGTTGCATTCTGGCACTGACCTG<br>GGAGGAAAATCAGCAAATCCAGCAAGTCTCGAATATTTGCGAAGCTTGTCCACAAAG<br>TGTTCTGATGTAGATTTCTTTTTTTGCCGTCTAAATAATGGTCACGATTGGCCCACCT<br>GGCATGATTGTGCTCCAGACTGG
promoter_TPM10000_04	ACTTTCTCCTTCATTTTTTGGTATTGACAACACTTCAAATCAAGCCACATACACAGTAC<br>ACCGAAAAGCCATATCCGGAACGTCACCCGTACAATCCACTTCTTTTTTCCATTATTA<br>TATGATACCTTTCGGGGCATAAAGGAATCAGCCCCTATACGCGTTAGTATCCCAAGTC<br>CATACATACACCCCCCGAACATACGAAAAAGCGTAACACATAATGCGCTACCCCCAA<br>AAAATCCGACCGTTAACCATTCCCCTTCAGGGAGGGAAAAATGGGGTGGCAGCCCC<br>GTCTGCCGTTTTTTCGCATTTCTACCTTTCCTCCCTCGGGTGAAATTATTTTCGAGAG<br>AGATATAAATTTTTAGTTAGTTGTATGATTAAGTTCTTTATTTAATTTATTTCTTACTAATC<br>TATTTTTCATTAGCAATACAAATTAAAATAAGTAGTTTTCTTGTTTTATTAAGTTAAGCAA<br>TTATATATAACAAACAAAAAACGAACAAAAA
terminator_TPM10000_04	TCATTCAAAAAATATTCTGTTCCCCACTTATTTGTTTTTTTAATCGCAATTCTCATCATT<br>TTCATATATTTTCACGTCTTATCTAGTTTGTAATTTTTATTTTTTCGTTCCATTTTTTTGT<br>TCTATGTTAAATAGTATTAAAAATTATTGTATATATTAAATATAATATAGATTTAAATCTCTT<br>TATTAGTATATAATTACCTGAATAAGATTATATCAATTATATATTTATAATTAACTTACTTGT<br>ATTTGTTAGTATTATTTTTTTTCTTTTCTGCTTTAAAGAAATTGAAGTTTTGCATGGTTGT<br>AGGAAGTTGGGTGTGGGTGTTGAATTTTTTGTGCTTGCAACCAGCTGGTTGGCTTT<br>TTCATTCGACTATCGCACTATTGGCTGTCAGCATTACACATGCGAAATTACAACAACC<br>AACACACACGAACGACTACAATCATCACACATCTCATCATTAATATAATGGGCGGAGC<br>GGACACCTTTCCACTCCTCAT
promoter_TPM10000_05	AATCGATCCTCATATTCCCTGGCGAAACACAAGTCCAGCCAATAACGCTCTTTGAAA<br>ATTTCGCTAAAACTGATACTTTGTTCGAAAAGTTCACTGCTATACAGTACTCCCCAGT<br>CCTCCTCTGCCTTGTTGGCAGTTACTATTCTACACTACACTTGTGGTTTAAAAACTCG<br>GGCTTCTCCTAAGATCAACATTAGTACTGAACATACTAATAAAGCAAGTAACCCACGC<br>GTCTACCCGTGCTTTGGCTGCCAACGCGAACGCTTCACACGGGACGGGCCAACCC<br>CAAGCGAAATTCCATTAGTGACTTCACAACTTTTGAAGAGAAAAAATCTTATTGAAAA<br>AGCTTGTATTTATTCATTTTTTCTTCCGTTCTTCTTCTATTATTTTGTTTTTTTTTCATTA<br>ATTACTAATTCTTCTTAGAATTAAGAACTTTATTTTAATTATACTTATTTAGTTAAGATACA<br>ATACTTAAGTACACAACTAATCACAATCAACAA
terminator_TPM10000_05	AATTTTTAACTATTTATATAATATAATTAATTTAATTAAATACTATTATATATATATATATTATA<br>AGTCATTTTTATATTATTATCGTATTTTTGTTTTTGAGTTTTGCGAGATGGGGTTGTCTT<br>CTAAGGTGATGGAAGGGCAGAATAAAGTGGCAATGTAATATTACAGTACTATAAGAAA<br>CAATCGCCTTGGGAGTGATGTGCGCTTTGGTGGCGTGTTCCTACAATTTTTATTAATT<br>GATCTGCTCGTCCCTATATTAAGCGCACGCAACTCATCCTACATTCTACTTCTTTATTT<br>TTTTCTCTGAATCTTGGGGTCGCATATGCGACGACCCCAAA
promoter_TPM10000_06	AAATATAGGATGGTCCACTTGGGGGAAAGAGGGTGGCCAACGATCTGATTTGCCAA<br>ACTTTTGAAATTCAATTTCCAACCAAACGGAAGTGGTGATTTTGTTTTAAGTTGAATTT

	TAATAATTTTTGCAAACTTAGTTTAATAATAATAATATTAACATAACTTCTATGACCATCTTGCTATCTACTTCCCTTAGTTCTTTTCTATTTCCTCACAAACTTAATACGCGCATTCTTTATTTCGTTTACCGCCCATGAGCAAATGAGACCGTGAAAACGTTCACGGGAGTGCACACCGTACCCCCTGTCAGAAGTAGTTTTGTTTGTCTTTTTAAATAATATTTAATATGTAAAAGTTGGTATGGTTTCTTGTTAGAACTTTATTTATACTTAGTTATATTTTTTTTTCCTCTACTATTTCTTATTTCATAGTTTTATTAGTTTTATATTTCTTTTTGTTCTACTTAACAACTCAACTTTTTACTATTACAAACGAACAAAA
terminator_TPM10000_06	ATCACATTAACATAAAAGGAAGATATTCTATGATTAAGGTAAAAATTTTCCCTCATGTATATATGTACTTTTTCGAATGATTTTCATTTTTAAAGGCTTTTCTTCTTTCCTAGTTAGTTATAATAATAATTTCAATTGTAATCAATTAGAGTGTGATAAGTTTAAGTTCAATTAAGTATAATTAACTAAGTATTAGTTAGTAATTATATATATAATTAAGTATAAAAATAAGATATATATTAACGATTTTTCATTGGCCTTATAATTTTTTTTGGTCATTGGGAAAAATGATGAAGTTTGCTTAAATTACATAGGGATTGTGTATTTGGCAGTGTTCATATGAGTCCAAAAAGGTGCTTTGGTCAATTGATACAATCCGGCCTCGTGGGCTTTTCAAATAGCTTGTTTAGCTTTCATTAAACAGCTAAAGTCATTTTTGGGTAGTAGTCTGAAATTTGCATCCCGTATCTGGGTCATGCAATGGCACCCATGTTTCTTGT
promoter_TPM10000_07	AATCGATCCTCATATTCGCTCCCGAAACAGAAGTCCAGTCAATAACGCCCTTTGAAAATTTCGCTAAAACTGATACTTTGTTGCAAAAGTTCACTGCTATACACTAGTCCCCAGTCCTTCTTTGTTTTGTTGGCACTTGGTATCCTACACCACACTTGTGACTTAAAAACTCAGGCTTCTCCTAACATCAACATTAGTACTGAACATACTAACAAAGCAAGTAACCCACGCGTCTACCCGTCCCTTGGCTGCCAGCGCGAACCCTTCACATCCGACGGGCCAATCCCAAGCGAAATTCCATTAGTGACTTCATAACTTTTGAAGAGAAAAAATTTTATTGAAAAAGCTTGTATTTATTCATTTTTTCTTCCGTTCTTCTTTTATTATTTTGTTTTTTTTTCACTAATTACTAATTCTTCTTAGAATTAAAAACTTTATTTTAATTATACTTATTTAGTTAAGATACAATACTTAAGTACACAACTAATCACGATCAACAA
terminator_TPM10000_07	ATAATTTTTAAGTATTTATATAATATAATTAAGTTAATTAAATACTATTATATATATATATATTATAAGTCATTTTTATATTATTATCGTATTTTTCTTTTTGACTTTTGCGAGATGGGGTTGTCTTCTAAGGTGATGGAAGGGCAGGATAAAGTGGTAATGTAATATTAAAGTACTATAGGAAACAATCGGCTTGGAAGTGATGTGCGCTTTGGTGGCCTGCCCCTACAGTTTTTATTGATTGATCTGCTTGTCCCTATATTAAGCGCACGCAACTGATTCTACATTTTACTTGTTTATTTTTTTTCTCTGAATTTTGGAATGCCATATGCGACGACCCCAAA
promoter_TPM10000_08	AATCATCATGGTGGATTAGTAGTTTAATTAAGCAATTAACTAGTTACTGATGACAACTCAGGTAACAAGATAAACAATTTAATTTAGTTAGGTTCTATCCTATAAAAGTTTTTTAAATAACCATGATCAAAACATGTTTGACATGCGATTTTCAACTCCATCTTTTCCCCAATTAACGCATATAAATAATCAACCATATACACCAGTCCGTACTATTATTTGCAGAATTATCACGAACATAAAAAAAAAGAAAGTATCAACACCACACATACCATCATGAAAGAAGATTCCTTCCCAGCCGGGTTTTTCTGACCAACAAGGATTTAGATAAATTGAAAAAGTTTTTTTTCGATTTTTACTCTATTTTTTTTTTAATTATTTTTGATTAATTTTGTCTTATAAAATTTACTTACTTATCATCTTTTACCTACATTTTACGTATTTAAAGCTTTTCAAAATAGTAGACATCAAGAATATCTCACAACATTCAAAAAATAACAA
terminator_TPM10000_08	ACATTAATAAAATAACTTTTATAATTCGTTTTTCTTTTTTTTCTTTTATAGTTAATTAAGTAATTTAATAAATTCGTTTTGATCGTAATTAATTTTTTTCCCTCTTTTTTTCATTAAATGAAGTTAGTAAGTTATAAAATAAAACTGTATATCTATTTATAGCTTTTTTAGTATGTAATTTTTTTTTATTTATCTTTTAACTACTAACAACAAAATTGTAAGACTGTTTAACTATGAAGCAGGAAAATAGGGTAGGAAACCAAATATATAGTGTTCTTCCGAAGTCTAATGAAATCACGGTGGCATGCGTGAGAGAGATGGTGTTCTTCCAAAATGAGATATAAAGGAGACCACAAAGAGAAACATGAGAAAGATAATTTGTCTATTGATTATTCTACTCACATCAGGGTTTATAAAAGAATGAATTCTCTTTATTCAACTTCGTTTATAACAACTAGTATTATATAGTTTTTGCTTTT

	GATCAGATAATTTGTTCGTGA
promoter_TPM10000_09	GTCGAGATGCAATGTTCTGAACACTTTTTACACGGTCCGACGATTCTTGTCAGAGCT GGACAAGGTAGTTAGTAAGTTGGTTAAATTTAGTATAGTATTTAAATTATATATAGTCATA ACTTTTAATCAATTTACTTTTAAATTTATAACTGTTTTTCCCATTGAACTATTCTCCTCGT AATTCTTCTCAAAAAAACAATATTTTATTTAATATTAAAGAGAAGCCCATCCGAACATCC ACCGTCCACCCAGTTAGACGGCAGGAGACGGGAGAGTGACTGACTTCCGAAGCCC CTGCCTTCCGCGGACTTATTTTTCTTCCCACGTAATTATAAATTTTAATAATTATTTTATT TTTTATATCAAAAATTGTTTTAAATTCTTTTTTTCATTCCCTTATTTTCATATTTACTTCTA ATCTTATTTTATAGTTTTATTAAATCTTTTTTATAGGTAACTTACTTACAAATCAACCAAC CAACCACTAAAGTCACACAAAA
terminator_TPM10000_09	AATTATTTACCTATTTTAAATTTTTTATAATTTTTTATTTATTACTTTCTTATTATATAAATTT AGTTATAATTTATTTTTATTATTTTTTATGTTTCTTATAAGTGTCGACTGATTACGAGGGG AGGTTTTCTTATTGCTATGCGCTTTGTGGTTTGAGGTGAAACAGTCGCGACTTGGCA GTTTTGGCTTCAACGAGCCTTAAGAATTTTTTTGTGGTTTTCTCACGGTCCGTTTTGA GCTATTATATGTTTAACGGCTCATTAGATATTCCACATTGAACCACACCGTTCACCATC ACTTTTTGGTTTTTTTCATAAAATTTTGCATCACTTTCAAAATGGAACGCTAAC
promoter_TPM10000_10	AGAAAACGAAGACACCATTTTTGAGCCTGCCATATTCCAAACTTCTTCTACCCACTTC ATAATCGACTTAAATGAAATTCAGTCATAAAACCGCAGAAACACACAGACGCGCCCA CCCGTAACACGTCATCTTATTCATGCCTGTATTCGACCTCCTCAAGCAACTATTTTGA CAATCCCCAAACAATCCAAACACCCAAAAGTAAACCCACCATTACACTTCCCCAACA TTCACACGGAAATTTTCCACCCTTCCCTCCTTCCTCCTTGCCTCCTCCTACCTTGCG GGACACCCCGCCCCCGCCCGCCTTGCTATCCCTCATCCCCTCAATTTAATTAAATATT GATAAATTTTTGTTCTCATTTTCTATTGTGTTTTCTTTTGTTTTTTTTATAAATACTTTTC CCGTTAAGATATTCTTCTATTATAAAGAAAAAGTTTTAGTTCGTTAATTATTTTTTTCGA CTAAGTATCAAATAACCATACAAACAAAAAACAA
terminator_TPM10000_10	AATTAATTTATTTATTATATCTTTTTTTTATTAACTATTTTTATCTTGTTTCCAATATTTATAT TTTAAATAAAGATTACTTTTTATTAATTTCAAGTCAAGTGAACGTGTAAGTTATGATTGT TATTTCCAAGCTCTCTTGCTTGCGTGAATGAATTAAAAGTAAAGGAATTTTCCGCATA CTAGAGGAAATTGTTTCTCGACAGCAGCGATGCGCATGATCCACAGACTGTTTTCTT AATCTTTCAATTGTGCTAATCAGTAATTTATATATGTCACGGTGTTAGCTGGGACAAAT TTTATTATTTGTTTTTTTGGGTTTCTACCTGGTAGAATCTATTTCTTTGGAG

Supplementary Table 7. List of DNA fragments used in constructs for the generative modeling experiment.

construct	sequence
promoter_TPM10_05	TTTAGCACGCGGGGTGTAACTCAACAGAAAAATGTGCCATAGAACAAGACTAGGCA<br>AAAGCCAAGGAGCGTTTGCCATGAACTTCCACAACTGCAACGCAACCAGCGCTGA<br>CGACATCCAAAGATCAAGAACTCACCCCATTCCAGGTTTCATAAGTTGGCAATATCG<br>ACATCTCAATTCCCAGCGGATCTGTACTGACTAAGCTGTCTGCAGGCTACAATCAGA<br>ATTAATTTCATAGAAAAATGATGGGGAATTTCATTATAGGTTAATTGCAAACACAATTAA<br>ACTTTGCAGATATTATGTCTTATTTCTTCTTAAAATGGACTCTATGATTATCGTATTTTC<br>CCACTATCGTAACATAGGGCACCGAAAAAATAGCGATCTGTATAGATTACGTGAGTTC<br>AATATTAGCTAAGCTGTGCACATCACTTTGCCGCTTTTGTTACCATCCGCTTCTCTAA<br>AATTGAATTAAAAAGGTGCTTTTGAAATTAATGCAGATTTTCGTCAAGACTTTGACCG<br>GTAAAACCATAACATTGGAAGTTGAATCTTCCGATACCATCGACAACGTTAAGTCGA<br>AAATT
terminator_ TPM10_05	GATCCCAACGAAAAGAGAGACCACATGGTCCTTCTTGAGTTTGTAACAGCTGCTGG<br>GATTACACATGGCATGGATGAACTATACAAATAGAATCATCTCTTCAAACCACAAATAT<br>TTAACGGCACTACAAAAAAAGGCGCAAAAACGAAAAAGTACTACAGCAGCCTTCAA<br>ACACATTCAAAAATACGCTCATGTGGATCTCTTTTTATCATCATAATGGGCGACTATTT<br>GTCCTTCTATTTATTTTTTTTCAGACTTCTGCATCCCTTTTGCGGTATGCCATATTGTT<br>CGTCTCCATAGACAAAGAGGGGGAGATAGAACCCATCACTATCCTGTTCATTTTCCA<br>TTTATTTTTTTTCATTGATTCGGCGTTATAGTAAAGAACATCAACAGGGAGGACATGT<br>CCAATATTTTACTGGAACGGCTTTGGTAAAAACTGGGGTTAGAGCCTGAAATTTTCT<br>CTTACCTGAATCTAAATAGTAATGATATGGTTGTCTGTTGTGGGGAAAATGTCAAACC<br>ATAAGTTTAAAAAAGTAAAAAACGATCTTCTCAAGAAGGAATAATATAAAGCATGATAA<br>AGTGAGCCTTGGCACACGACTAGCGCTTTCAGATATTAAAAAGTTTAGATGTAGGTT<br>TTAGCGGTAACAGTTATATAAATCGTGTTTCTTCTCTTGATGAAA
promoter_ TPM10_06	TTTAGCACGCGGGGTGTAACTCAACAGAAAAATGTGCCATAGAACAAGACTAGGCA<br>AAAGCCAAGGAGCGTTTGCCATGAACTTCCACAAACTTCGTATAAGCAATGAACAAT<br>CCCAAAACATACAGTAAAGCGATTCTATTATATACGGGAAAGAATATCACTGCGCATG<br>ATAAAAATTTTACTGCGATAGCAAGATTCATTAATATTTTTCAACTTCTTGATTGGCCC<br>TTCGTTGTAATACAGGTGCAATGTGCACATCATTGAGGCACAAATAGGTAAAAAGTC<br>CAAAGTGGATTCTTCTGCTGAGTGGTTGATTAATAAATACGAAGCTTTCTGAACTGAT<br>TCCTCAGGTTCCAATTTTCCCATTGCTTTTTCTTTTTTTCTAATATTTTGTTATATTATTT<br>GAGTTATGCGATCATTGTGCATCTCCTTCAATTTACTACACTATTTTTTTATTAAAAACA<br>ATGACCACATTCCCACGGCACCTTACCAATACAATGCAGATTTTCGTCAAGACTTTG<br>ACCGGTAAAACCATAACATTGGAAGTTGAATCTTCCGATACCATCGACAACGTTAAG<br>TCGAAAATT
terminator_ TPM10_06	GATCCCAACGAAAAGAGAGACCACATGGTCCTTCTTGAGTTTGTAACAGCTGCTGG<br>GATTACACATGGCATGGATGAACTATACAAATAGAAATTATTGAATATCGGGAAAAATT<br>TGCATAACTGAATAATTGCATATTTTCAGCAAATAAAGAGTAGGAGCGGTAGACTCGA<br>AAAAAATACAACATAAGTTTTTGATATGTATCCCAACCACTTGCATTCAAGTCTTTTAT<br>ATGAATGTCAATATGTGTTGACTTAAAGAATCGGATAGTATCTTATTCTTGATCAAGGA<br>ATAATAATATTTTCTGTCTAAAAATTAAGTTCAGAAAGCGTCCTTTTGGTCACACGACT<br>AGCGCTTTCAGATATTAAAAAGTTTAGATGTAGGTTTTAGCGGTAACAGTTATATAAAT<br>CGTGTTTCTTCTCTTGATGAAA

promoter_ TPM10_07	TTTAGCACGCGGGGTGTAACTCAACAGAAAAATGTGCCATAGAACAAGACTAGGCA AAAGCCAAGGAGCGTTTGCCATGAACTTCCACAACTTGTTGATAATTAATTCGTTGAT GCAAACCTCTAAAAATGAATTGAAAAAATAATAATTTAAGCAGTGCTCGCCTTGAGAC TTGGAAGAAAAAAAAATTTAATAGAATGAAAAAAATTTCGTGTATGTCTCTATAGTCATT TGCGTCTCCTGCGTTTATGAACCTAGATTAATATGTAAGTTAAAGAGTATATCTAGAAA ATAATTGTATCAAAAGAATCAATAGCTCTATATCACTGTTTCTAATTGTATGATTTTTATA TTGAGAAAAATCACCATTTTTATTTCTGATGATAACATTGCAATGTAGAAAAGCGTTGT TTTCAAAAAGCGCAATAAAAAAATCAGAAAACCCACTATGCCATTCAAAAAATAGAAA GAATATAAACGAATTCAAATAGTCCAATGCAGATTTTCGTCAAGACTTTGACCGGTAA AACCATAACATTGGAAGTTGAATCTTCCGATACCATCGACAACGTTAAGTCGAAAATT
terminator_ TPM10_07	GATCCCAACGAAAAGAGAGACCACATGGTCCTTCTTGAGTTTGTAACAGCTGCTGG GATTACACATGGCATGGATGAACTATACAAATAGAAAGGACCGATGGCAAGTCGTAT TTTATAGCATTCTTTCGTGATTTTTTGTCATTCTAGTTATAATTGGGGCCGTGGCCAT TTACGTGTGTGAACCTACGTGCCAAAATTTAACCCCGAACATTAATATATATAAAAATA ATAAAACATAATGGTCTTGGTAATTTTCCATATTTAAGTAATTTATTTTTACCTTTGTTTA ATACTGACTTTGAAACGAAAAAATTAAATTTAAGATAGATAAGAATTAACACACGACTA GCGCTTTCAGATATTAAAAAGTTTAGATGTAGGTTTTAGCGGTAACAGTTATATAAATC GTGTTTCTTCTCTTGATGAAA
promoter_ TPM10_10	TTTAGCACGCGGGGTGTAACTCAACAGAAAAATGTGCCATAGAACAAGACTAGGCA AAAGCCAAGGAGCGTTTGCCATGAACTTCCACAACTTATCTACAAATTCCGCAATCC CTCCAACCAATAAATTTTTTTCCAAAATTAACTGATAACGGTAGTACTATAAATAAGTTA AATATCAATAAAAAATTTTGAATGTCCTAGAAGTTTTCTATTTTTGCTAATTATAAAAATC CTGATATAGATTTTTGTGGAAAGGAATTCATGCGTGTTTAGTTCTACATTCTGTAGTAT ATTCATGTATATATATTTAATCGCACCCATGCGCTCTTTCAATCACAATCCCCGATTGA TTTGTATCTGTAATAAATATTTAAAAATCCTCCTACAAATAAGTGAAGTAATTAAGCAAC AAGGAAAACTCCATAAAAAATGAAGATCATATTATATTCACCTCTAATTAAATCGTAAC AAATCCTCACCATTTGTATATAATGCAGATTTTCGTCAAGACTTTGACCGGTAAAACC ATAACATTGGAAGTTGAATCTTCCGATACCATCGACAACGTTAAGTCGAAAATT
terminator_ TPM10_10	GATCCCAACGAAAAGAGAGACCACATGGTCCTTCTTGAGTTTGTAACAGCTGCTGG GATTACACATGGCATGGATGAACTATACAAATAGAAAATTGTACAGTTGCACAATGTT TTCTTCTATGTCGGGTTGGCATCATCGCGAACTATTGGCAGCCCAGCAAACGCTATC TCGATATGCAAATTAAGAGTTTGTGATAGCTGGATCAGATATTATACTATTTGAAATTTT CTTTACTATCATGGTTCTGTTAAGTGAATTACTATTGATTCAACATAATGCAACGTTGA GTTTGAAATGTCTTTAGTGAGAGCATAGCACTTAAATGGAGACAAAATATACACACGA CTAGCGCTTTCAGATATTAAAAAGTTTAGATGTAGGTTTTAGCGGTAACAGTTATATAA ATCGTGTTTCTTCTCTTGATGAAA
promoter_ TPM100_01	TTTAGCACGCGGGGTGTAACTCAACAGAAAAATGTGCCATAGAACAAGACTAGGCA AAAGCCAAGGAGCGTTTGCCATGAACTTCCACAACTACTCAATTCGCACTTTAGCAG TGTAAGTAAGAGCGACATATACGTTGCTGTAAATGTATGTATAGGACTTAAATGGACA CGACCAGGCTTTTGTGCAATGAATTAGTTAAGCACGACAGATCGTACATAAAGCACC CCTATTTAGCTTTTTCACCACCAGCGTCGAGTTTTATAAAGGTTCCTAAGCTCAGATA ACTCCTAAACCTATATCACATCCAGTCATTTCTAGCTTCCTTCTGGCAAATCAAAGCC CCCCCCCAACAAAAAGTTTTTAAGCATTGGATTCCGGACCCCTTCCGAGCAATCGT GAAATCCGAGGCAAATAAGGTAACATTCCTGAAAGCAGGATGGTTAAAAATGGAATA TCGCAATCGAAGTAGAAATTGCAAGAAGTATCTATAATAATGCAGATTTTCGTCAAGA CTTTGACCGGTAAAACCATAACATTGGAAGTTGAATCTTCCGATACCATCGACAACG TTAAGTCGAAAATT
terminator_ TPM100_01	GATCCCAACGAAAAGAGAGACCACATGGTCCTTCTTGAGTTTGTAACAGCTGCTGG GATTACACATGGCATGGATGAACTATACAAATAGCTTAAATTTAAATGTTATATATTTTT CTGCTGTCTTTACAATTGTCTTTTATCTTGTTAAGGCACCGAGGATATGATGGAGGAA GTATAAAAAAAATAGAAAAGCAGTGGAGATAGGATAAAAATGTCGAGATGGAGAAAA TTTCAAAGCAACAAAAACTTCGATGGGAATCGAATGAAGCGCCGACTTTCCTGACAT CACGCCCTACATTTATCACCGTAGAATGAGGGAACCTGGGCCACAAAATTGCACAC ACGACTAGCGCTTTCAGATATTAAAAAGTTTAGATGTAGGTTTTAGCGGTAACAGTTA TATAAATCGTGTTTCTTCTCTTGATGAAA

promoter_ TPM100_03	TTTAGCACGCGGGGTGTAACTCAACAGAAAAATGTGCCATAGAACAAGACTAGGCA<br>AAAGCCAAGGAGCGTTTGCCATGAACTTCCACAACAGACAAAATTGCAGTCAAATAA<br>GTCCTTCTCAAGTACAACGCACTGAAATAATATCTTAGTAATTGAAACTATGATGTAAC<br>CACCTTGTTAGAGTCTTGATATTTATAGCGGGATGTATAAAACAGCATAAAAAAATGAT<br>TTTTTTTTGAAGGCTGTATTATCTTGAAATTTTTAATATTTTAATGAGAGAAGGTCAAAA<br>AAACAAAGTAGGACTTTGAGATAAGTTTTCACATGTAAATATTTCCCCTATACAACAC<br>GTCATTGATCGACACCCATCTTTTTGTTCTCAAGTACGTGGTTTGCCAGCTACAACA<br>CGGTCTCAGCTTTCTTTTCCAGGCAAACAAAAAAGAAAAAATAACAAAATAAATAATT<br>ATTCATTGCTATTATACTTGTAAAGAATCATACTCTCATAATTCCACACTCGCTTGTTTC<br>TGAAAGATACCACAAACTTAAGTCACTAAAAAAATGCAGATTTTCGTCAAGACTTTGA<br>CCGGTAAAACCATAACATTGGAAGTTGAATCTTCCGATACCATCGACAACGTTAAGT<br>CGAAAATT
terminator_ TPM100_03	GATCCCAACGAAAAGAGAGACCACATGGTCCTTCTTGAGTTTGTAACAGCTGCTGG<br>GATTACACATGGCATGGATGAACTATACAAATAGACAACGTCAGCTCCATCAGCCTC<br>TTGAACCTCACAATCATTTTTTTTCTTCCGTGCAGTTCCTCAGCGCCATTTGTAGAAT<br>CTTTGTCTAAAAGATAAAAAAAAATCGCAGCTGCTATAATGCAATTGAACTCTAGAAA<br>AACCTCGTATCTAAAATCATCTAGGAAATATAATCATGATTGAATATATATTGCTTGAAA<br>CAGCGGATACAGTAATTAAAGGTAGCGTTGGTAGCGTAGTTTTTATTACTTTGGATTC<br>CGTGGCCACACGACTAGCGCTTTCAGATATTAAAAAGTTTAGATGTAGGTTTTAGCG<br>GTAACAGTTATATAAATCGTGTTTCTTCTCTTGATGAAA
promoter_ TPM100_04	TTTAGCACGCGGGGTGTAACTCAACAGAAAAATGTGCCATAGAACAAGACTAGGCA<br>AAAGCCAAGGAGCGTTTGCCATGAACTTCCACAATTCTGTCTTCTAGGGAGCATGG<br>GTAGCCAGGCAAAATACCACACTCTAAAAAACCGCAGTGAGAAAGAGAAGGCAGG<br>GTGGAAAGCTCAAATGAGGCCAATAACAAAAAAATTTGCTCCACGACATACCCTCGT<br>CTTTCTCTAACTATTGAAGAAAAGGTGTAAATTTACCGGTATTGCCTTACGATATAAGA<br>ACCCGGCAAATCAACACTAATTGTAATTGGCTGTTTCCTTCAATAGTTGAGTTATCGT<br>GCTGTCCTTACTAAGTAAAAAAAACATTATTCGTTTTTCCTCTAAAGAGCAGATGTCA<br>GCTTGTTGCAGTGATTCTCAATTGGCGATAGATTTCTTACTTGCTTTTATTAACTAAGA<br>ATTATCGATCCAACCTCTAAACAATAAGGAAAAAAAATGCAGATTTTCGTCAAGACTT<br>TGACCGGTAAAACCATAACATTGGAAGTTGAATCTTCCGATACCATCGACAACGTTA<br>AGTCGAAAATT
terminator_ TPM100_04	GATCCCAACGAAAAGAGAGACCACATGGTCCTTCTTGAGTTTGTAACAGCTGCTGG<br>GATTACACATGGCATGGATGAACTATACAAATAGTATATGTGGTAAGTTGAGCCGAAT<br>TCCTTCTATGTTTGTGCAGTTTTGACGCTTATGTAAGGAGTGTAAACATTCTAGTTGT<br>AGGTCTACGGGGCGCTAGTCTGTATCTCTCTGGCTTAGTATCGTTAATTTTCTATGAT<br>CTTCGTTACTCCTTCAGTGGTTATACGTCATATGTATTCAAGAAGAGTGTTAGATAGC<br>TATATGCGTCGAATTAACTTATTATCTATATTTTCTTTTTTGTCGATCTTCTTGATTTAGC<br>ATATTTTAAATTTGAAAGTGGTCTACAGTGAATAAAGAGAAGGCAAATATAAAAGAAG<br>ATGCTTGGCTTTTTTTTTGTCGGCTTTTGTATCTACTAATTACGCATCTTAGTTCACAT<br>TATCATTTGGTAGAACTATACGAGATGAATCCAACAGCTTCTTCGAGGTTCAGATAAT<br>TAATAAGCTTTGATACCACATGAGAATCGTCCAATTTGTCTCATAATGGGAGCATTCG<br>ACAATGCCCACACGACTAGCGCTTTCAGATATTAAAAAGTTTAGATGTAGGTTTTAGC<br>GGTAACAGTTATATAAATCGTGTTTCTTCTCTTGATGAAA
promoter_ TPM100_05	TTTAGCACGCGGGGTGTAACTCAACAGAAAAATGTGCCATAGAACAAGACTAGGCA<br>AAAGCCAAGGAGCGTTTGCCATGAACTTCCACAAAACTAGCTAAGCAAGTAAAATCA<br>ATCCATGAATTTAATATTAATTTTTCCTTTCTAAGTAATGACTAAATTTTCTTTTCTTTGA<br>TCTTTTCCATTTAATGATTTTTTCTTAGTTAGTTTTTCTCGTTTTACTAACTTTTATTTCT<br>CCATCTTTTCACCCATATTTTTTATAGGCGAAATCACAAGTGTGTTCCACTTTTTCTC<br>GTGATGAAGTTTGTTGAACTACTTTTCTGGCTGATGACCTCGACACAACCAATCATC<br>CCCCAAAGAGCTCTTCCAAGAGTCCCGTAATTTACATGTTTTTTGTGTTAATTTTTTTA<br>AGCAAGCAATTATATACCCCTTCACCATATTGTCTTTTCTCTACAACAGTTTCAGATAT<br>CAACAAAGTGGAAGTGTCAAAAAAATGCAGATTTTCGTCAAGACTTTGACCGGTAAA<br>ACCATAACATTGGAAGTTGAATCTTCCGATACCATCGACAACGTTAAGTCGAAAATT

terminator_ TPM100_05	GATCCCAACGAAAAGAGAGACCACATGGTCCTTCTTGAGTTTGTAACAGCTGCTGG<br>GATTACACATGGCATGGATGAACTATACAAATAGTATTTACACTACGAGAAAGACTGA<br>AAAAAAGAAATAATTAATTCTGCTTTTAATTTTACATTTGTTAAATATTTATTTTTTCGTT<br>TATTTATATGTTTAAGGTTAGTATATTTCGAACAATATTTGAAACATAAGTAATCCTACTC<br>ATACGCCCCTCTTTACATATATGAACTGTAGAAGGAGACGTGCATATTTGTACATAGT<br>CATGGGACTAAATGGTAGCCAAATAACGAACGAGTAGGAATTGCACCCATTTCCAGG<br>TTGAATGGGGGTGGTATTTGAGTCAGCCGTCCATATTTCGTGCTTGCTTGACTTGTT<br>AAAGTAATTGAAACAACTAGAAAGAAATAGAGCAAAAGTTACAATATTTTGTTGATCA<br>ATTTCCCCATGATGGAAACATGTTAGTGAGCTATTGACATTGTTTTATACTAACTGTTA<br>ACTACTAATTATCTATATCCAATCATATAGATATCAATATACAAAGTTTGTATAAGATTAA<br>AAGTCACACGACTAGCGCTTTCAGATATTAAAAAGTTTAGATGTAGGTTTTAGCGGTA<br>ACAGTTATATAAATCGTGTTTCTTCTCTTGATGAAA
promoter_ TPM100_06	TTTAGCACGCGGGGTGTAACTCAACAGAAAAATGTGCCATAGAACAAGACTAGGCA<br>AAAGCCAAGGAGCGTTTGCCATGAACTTCCACAAATCCAAAAGAAGAAAGAAAATC<br>GAGTGAGAGCGCCTTCTATAGAGTGTAACTGCACAGACTCCGCGGAACTAAAAGAG<br>GCATTGGAAAAATGGTAAAAAGAGAAATATTGCAACAGCGTCCATTAGACCCTCTGG<br>ACGAATTTTGACTCAAGAGAGCATAAAAATTACATCGAATACGTAAAGAAAGTTCTGT<br>TGGTTTTTTTATAATCCTGTACTTTTTTCATGAAACTCTCGCAGCATTGCAAAAGTAAT<br>CTTGGGATTTCTTTTGCTTCGATCTCAATGGAATTAGTTATGGTCTTGCTATAGCCGC<br>GGGACAAAAAAAAAAGTAAATATCGATGAAAGGGTTGTTAAAGTTAAAAGGAGATTAA<br>GTAATATTTGTGATGAAAGTTACTTATCGAGCAAATGCAGATTTTCGTCAAGACTTTG<br>ACCGGTAAAACCATAACATTGGAAGTTGAATCTTCCGATACCATCGACAACGTTAAG<br>TCGAAAATT
terminator_ TPM100_06	GATCCCAACGAAAAGAGAGACCACATGGTCCTTCTTGAGTTTGTAACAGCTGCTGG<br>GATTACACATGGCATGGATGAACTATACAAATAGCGGCATTGAAGAAGTACATCTGAC<br>GAAAATAAAAAAAAAATTGTGTATATATATTTAGGTGCCGTTAACCAGCAGCACGCTGA<br>TGGCAAGTTTAACTGGGGAAAAGATGTTAGGAACATTTTGTCTGGTAGACAATGAAA<br>AAAATAATACACATATTTATAGTCACACCCTACAATAATTGGTCATAATCATATTATTTTA<br>TTCATAATATAACGCCAGATCATAATTGTTTTTGTGAGAATAATTTGAAAGGGGTACAT<br>CCATCTAGAACACACGACTAGCGCTTTCAGATATTAAAAAGTTTAGATGTAGGTTTTA<br>GCGGTAACAGTTATATAAATCGTGTTTCTTCTCTTGATGAAA
promoter_ TPM100_09	TTTAGCACGCGGGGTGTAACTCAACAGAAAAATGTGCCATAGAACAAGACTAGGCA<br>AAAGCCAAGGAGCGTTTGCCATGAACTTCCACAACAGCTACTGTATATACTGAAAAC<br>ATTTGTTTTAATTACCAAAAAGGTCTTATTTTGAACAGATAATGAGCTTTCCATTTGCA<br>AATGCTCAATATTTGTGATCCTGTTGATATCTTCCATCTTGAAACCTTGTATAGTGATA<br>GTATAGATCTTGCTCACATTCAATTTGAAAAATACATACACATTTTCTCTAATTCAATAC<br>AAGTTGCTGGAATCTTCGCAAATGTATGAACAATCAAGGTGACACAGTATTTCCTGA<br>TATAACAAAATAGTTTTCTAAGATTATTTTTTATCCCATTCGGCAACCTTAAGAGGTGT<br>TCCAAACGAATGCAGGTGACGGTTGTCTGACCACAAGGGAGAGATAAAGACAATAA<br>AAAGGATCACCCAATAAGAACTTTAGATCATAAAATTTTTTTAAATCTAAAAAAAAACA<br>CATACTTAAAATTCGTTTAATAAAGATATCATAGCATTAAAAAATTCTTCCTAATCAACC<br>TGTGAAAAATGCAGATTTTCGTCAAGACTTTGACCGGTAAAACCATAACATTGGAAG<br>TTGAATCTTCCGATACCATCGACAACGTTAAGTCGAAAATT
terminator_ TPM100_09	GATCCCAACGAAAAGAGAGACCACATGGTCCTTCTTGAGTTTGTAACAGCTGCTGG<br>GATTACACATGGCATGGATGAACTATACAAATAGATTCTTTACATTTTTTGATGTCCTT<br>CATATATATATGGACATTGTTTAATTTTTTTTACTTTTCTTATATAGATTTTATTGTATGCG<br>TGGATTACCGGGATGGAGTTGTCAGTGGTAATATCCATAGTAGTGGAGGTAAAGGAA<br>AGGATGTAACATATTAAAACATTTGCGCAATTAAGAACATCTTTCTATTGGGCACACTT<br>GTATACTATATTTATATATACAAAAAAAAACATTGACTTGATATCAGGTTATTACTAATTT<br>GATATTATCGAATTTTTACTACTAAAATAATCCACTAAGGTGAATTTTAATAAAGGTGG<br>GGTCTATCTATGAACACATGAAAACAAGATAGCTCAACATAGTATCGAAAATAACTTA<br>GGCTGGCATTAAGTGCTATAATAAAAGAAAGCAGACCTTTGAAACCGGGGAACGAC<br>GAATGTGAAGAAGAAACATAAAGCACATCACATTATCCTCTCAAGCGTTTATTGAAAG<br>GGAGCCACACGACTAGCGCTTTCAGATATTAAAAAGTTTAGATGTAGGTTTTAGCGG<br>TAACAGTTATATAAATCGTGTTTCTTCTCTTGATGAAA

promoter_ TPM1000_02	TTTAGCACGCGGGGTGTAACTCAACAGAAAAATGTGCCATAGAACAAGACTAGGCA AAAGCCAAGGAGCGTTTGCCATGAACTTCCACAATTGCAGCCGGTGTAGAGTTATT TTCCATACTAGATCCGTCACCCTGTTTTTACGCGCAAGAAGCAAACCAAGTTAAATA CTTCACAAGTAACCGCCACATAAAGTACAGAGCTAAACGAACCCTCACTATTGGATT CGTCGGCCCTGCCCTATCGATGAGCCATCAGCCGACGGCTTTTCAGGACTGATCGT ACATTAACGGCAACCACGTCCTCCAAAACTGTGTTTCTTTCTATTGACGATGTTGTG GTGTATCGGCAAGGTTCTTCTTTCCACCATGGCCAAAGATCGCATCAGGAAAGAGC GCACTGGCCGAGAGGCAAGACCCCGTTCTTACCAAACTTCCTCATATAGATATGTAG CGGATTTAGGTATTAAACAAGTTATATCTTTCTTTCTTACCAAACAAATGCAGATTTTC GTCAAGACTTTGACCGGTAAAACCATAACATTGGAAGTTGAATCTTCCGATACCATC GACAACGTTAAGTCGAAAATT
terminator_ TPM1000_02	GATCCCAACGAAAAGAGAGACCACATGGTCCTTCTTGAGTTTGTAACAGCTGCTGG GATTACACATGGCATGGATGAACTATACAAATAGGTTTATTTTTTATCCAGTTTAATTTG TGTTGTTATAAAATATATATGTTTATTAATATTTAGTATATTTTATGTATTTTTATTTTCATT ATTGCCTTTTCCTTTTTTTTCGTTATGTCCTCGGGTTGTTTTTTGCTCCTGTAGTCCC GCGGTGTGGTGGTTGTGACACTATTAACGCACTGGCAGAAAAAATTCAAAGACTAG CCTGGTATTTTTTCGAATATTCATTTAAATCGTTCACATCTACAAACTTTCCGACTCTA GTTTTATCTGACAACATCGTACTTAGCAAAGTTTTGGTGACCAGCAAATGTAGCAGA ACAATAATTGAGCCACACGACTAGCGCTTTCAGATATTAAAAAGTTTAGATGTAGGTT TTAGCGGTAACAGTTATATAAATCGTGTTTCTTCTCTTGATGAAA
promoter_ TPM1000_03	TTTAGCACGCGGGGTGTAACTCAACAGAAAAATGTGCCATAGAACAAGACTAGGCA AAAGCCAAGGAGCGTTTGCCATGAACTTCCACAAACCTTTTTGAGACGCCTATTCA CCCACTGCAATCGCACACTAAGCTGTCCAGCCTTTAACTCCGTAAGTCCTCTTCTAT TTCCCACCATCCTATTATCCCGTACGCCCGGTCGGGAATCCAATGATATCTTCGTAG GCGCACGCGCAAATCAGTACTTCATTTTTCCTTTTCAGTGTAGCATCCATTTTCCTCT TAATGCCTTCCAATCCCCCCATCGATGATCGCAAATGAGCTCCTTCAACCTGGCAAT GCTTGGGCGAAATCCGCAAACCCTCCTTTCTACCCTATTGCGAAGTCTTCCGCCTT GTGAGCAATCACTAGATACGATTTTCACGATTTATTGCTTTTGTATGAAACTCTTTTTA GTTCTTTTCTGTTAGTATTTTTATTCCTTAGTAGAAACAAACAACCAATGCAGATTTTC GTCAAGACTTTGACCGGTAAAACCATAACATTGGAAGTTGAATCTTCCGATACCATC GACAACGTTAAGTCGAAAATT
terminator_ TPM1000_03	GATCCCAACGAAAAGAGAGACCACATGGTCCTTCTTGAGTTTGTAACAGCTGCTGG GATTACACATGGCATGGATGAACTATACAAATAGTTAGTTTTTATATAGAAAAACTATTG AAAACTATAAAGAGCAGAAAAGTCGTAGAATTAAGATGATTAATAATTTCTTGTTTGTT TTTGTACTTTTTTTACTTTTGCCAATTTTTGGGTAGGGACAAAATTTGTTGCAGGGAG AACAGCATTCTGGTGAATTGTGTCCACCTAAGTCTTCGACGATGAGCGAAGAGGAG AATGGCCACCCATGTAGAAACTATTTCACGCATAATAATTCAAACATAGATAACAACTA TTTATGTCCGTGAGCGTGGACTGCAGAATTTTTATAAGCAATCACACGACTAGCGCT TTCAGATATTAAAAAGTTTAGATGTAGGTTTTAGCGGTAACAGTTATATAAATCGTGTT TCTTCTCTTGATGAAA
promoter_ TPM1000_04	TTTAGCACGCGGGGTGTAACTCAACAGAAAAATGTGCCATAGAACAAGACTAGGCA AAAGCCAAGGAGCGTTTGCCATGAACTTCCACAACAACGCGGAGGCTATTGCCTCT GCCGCTCAACCCTTCCTGGCTTCCTTTTCCATTTCACAGGCGCTGTCTGAATAGGTA GGTGTGAATGTTTCACCCAAAATGTGCAATGCGCGTTGGCAGATTGTCTGGTTACC TTTTCACTGGTTGTTTTCCTCTTGCATCCAAATTCACATACTACTCGTTCATAGTCATT CAGCACTACCAACCAATTTTTTTCCTTTCCATTCTCCGTTCGTCACTCCTCTTAAGCG TGTGCTGCTTCCGCAGGCCCTCCGGCTGGGACGAGTGGGGATGATGGCCCGCCC TCCTCATTGCATAAAGTCGGAAATATAATATTCCTTTGCAGGTTTCCTTATCTTTTTTG ATGCGTGCGTTATTCTTCATTTTTTCACACGAAACATTTGTATAGATCACAACAACAC AAAAATGCAGATTTTCGTCAAGACTTTGACCGGTAAAACCATAACATTGGAAGTTGA ATCTTCCGATACCATCGACAACGTTAAGTCGAAAATT

terminator_ TPM1000_04	GATCCCAACGAAAAGAGAGACCACATGGTCCTTCTTGAGTTTGTAACAGCTGCTGG GATTACACATGGCATGGATGAACTATACAAATAGATATCTGGAATCCACTCCGCATCA TATTTATAAACTTAAAGTTATTTCGAGACAGTTTCTAATTGATTACTAGTTACTTATAGG TAAAGGGTTACCTTCTCATATATTTCCTTTTTAATTATGCTTATAGTCTTATATACTATTAT TTTCTTTTCGATTATAAGGTGTTTAATATATGTAAAGCAAATATATTACGGTATAAAATAA TAGTCTTTTTATAGTACATGTTTTTTTTCGCAATGTTTTTATTCTTCTTTGCTTTTTTCTT TCCGCCTGAGTGCTTAGGTGTTAGTTCAACGGATTTTGCATTACAGTTTTTAAAGAAT CTATTGCCAATAGGGGGCATATGGCTAGATATGAATGTCGCAACATGTCATACTATTA GAACTTTTACTTTTCGAGCTGTTTGCTCTTTCTTCTTATATGTAATCCACTCCAATGGT ATCACATTATTAGGCAGCATGCAAAATGAAAAAAAAACACACGACTAGCGCTTTCAG ATATTAAAAAGTTTAGATGTAGGTTTTAGCGGTAACAGTTATATAAATCGTGTTTCTTCT CTTGATGAAA
promoter_ TPM1000_05	TTTAGCACGCGGGGTGTAACTCAACAGAAAAATGTGCCATAGAACAAGACTAGGCA AAAGCCAAGGAGCGTTTGCCATGAACTTCCACAACCAAGGTTGCGCCTGCCCTCG CTCATCCCAATTTCGAGAACTTATCAAATTGAGCACATTTCACTATGGATACTGTCAG AGTCAACAATACACCCCTTTCAAACCGAACTGAAGACTTCAAATCAACTATGGAGAA AACATACTGTCAGTCACAGGCACAGCGATACTATCCAACATCCTATTATACGACATCT TATTTTTCATATTTCCATCCCTTCTTATCATTACTCCCTTCCCTTCCGTCCTCCTTTTTC CCTGCTGATTCCTCCCGGGGGCCACCCCCCGGCGGGCATGTTGGAGAGAGGCGG TCCCTGAGAGAATGAAAGGTAAAGATACCTGCCTAATCATGATTTTCCATTTTTCGTG CTTCCTTCCACCTAACAACATATTGTATGAGCTGTAAATTTTTCCATAACTTGAGTTAA ATTTTATTAAATTTTTTCATATTTTTTCCCCATCTCAAGTAAATAATCTCATTTAAAAATA ACAACAAAAAAAGAATGCAGATTTTCGTCAAGACTTTGACCGGTAAAACCATAACAT TGGAAGTTGAATCTTCCGATACCATCGACAACGTTAAGTCGAAAATT
terminator_ TPM1000_05	GATCCCAACGAAAAGAGAGACCACATGGTCCTTCTTGAGTTTGTAACAGCTGCTGG GATTACACATGGCATGGATGAACTATACAAATAGACCTAAATTTTCGTAGTTTTTTCTA TTAAGTATGATATTTATTCACGGAATATATTCTTATAAGTCGATTAGGTGCAGGAAAAAT GGCATTTTTGGAGAATATCATAAAACATAAAAAATTGTACAGAGACGGAATAGTGAAT TGTGGGAAAGCAGGGACAACAACGGTTTTCTTGCTCGCAATGAGAATCTACTCCTG GCAACTTTCCACAACGAACTTTTCAAGCCCCGCTATCACTTTGACCACCCCTGCGTA AGACCTGCTGCCACACGACTAGCGCTTTCAGATATTAAAAAGTTTAGATGTAGGTTT TAGCGGTAACAGTTATATAAATCGTGTTTCTTCTCTTGATGAAA
promoter_ TPM1000_07	TTTAGCACGCGGGGTGTAACTCAACAGAAAAATGTGCCATAGAACAAGACTAGGCA AAAGCCAAGGAGCGTTTGCCATGAACTTCCACAATGAATGCCATTGTGCCAACCATT TGCATTACTTACTATATACAGCAGTAAGCCTGATTTCAGGAGGGTACAATCCACAAGA TATCCTAACTATCGAACAGATATTAAACTGTCCTTACATCATTAATCACCTTAAAAAGA GTGTTCCCTCTCCATATTATTAATTGTTCCCCCTGCTCCGTCTCATAAAACCATCACT TCAACAACTACAACAGCAATTAAGCAGACTTTTCGTCACCTTCCTACCCGGGAAACT TTGGGCAGATTTTATGCGGCCAGCCGTTTCTCACATTGTTGTTGTTGCGGGGACTC ACCAGCGGTTGGAAACTTGAGTTTTGAATAAAAACTATTGAAAAGTAGCTATATAAGA AATGAATAAAGCTTTTCTTAAAGTAAATTTAAATTGTTTTATTCCCTTTTAAAAAGTATTT ATATTTTCCTAGTTATCTTCTTTTATTTTTATTTTTATATTTTTCTAACGACCATAAAAAC GAACAACAAATGCAGATTTTCGTCAAGACTTTGACCGGTAAAACCATAACATTGGAA GTTGAATCTTCCGATACCATCGACAACGTTAAGTCGAAAATT
terminator_ TPM1000_07	GATCCCAACGAAAAGAGAGACCACATGGTCCTTCTTGAGTTTGTAACAGCTGCTGG GATTACACATGGCATGGATGAACTATACAAATAGATAATCCTATTATGACCTATTTATTT CTTTGTTTTTTCTTTTTTTTGTTCTTTTGTACTATAAGTATAGTAATGCATTCTTGTTTTT TTAAATGCAGTTCAAATATAAAATTTATGTGACAGAATGGCAACATACTCATGTTATTC ATTCAATGTACACACACACTTAAATCCACGAATCGCGAAAGTTATTGAATTCGCCCGA TCGCCTTCTATCATCTGTGAAATTTTTTTTGCAATTTCCCAAGCCACTTTTTTCCTCA CACGACTAGCGCTTTCAGATATTAAAAAGTTTAGATGTAGGTTTTAGCGGTAACAGTT ATATAAATCGTGTTTCTTCTCTTGATGAAA

promoter_ TPM1000_09	TTTAGCACGCGGGGTGTAACTCAACAGAAAAATGTGCCATAGAACAAGACTAGGCA<br>AAAGCCAAGGAGCGTTTGCCATGAACTTCCACAAGGTCTACTGTAATTCATTTTAATA<br>CCTTTGCTCGTTACCCTTTATCGATCCGCATGTACCGGCGATTCTCTCTCAATCAGT<br>GCTAGCACGGCACTGTTAAACATGATTGGTTCATCCCGATCCGCATTGTGCGTAGCA<br>GCTGCCTCGGTGTGATTCCTTAACTCAATCTACTCCCACACATCTCTTCATTTGTACT<br>TCAAGAGACCGATGAGCAGTAAAAAGGTATCGGAGAGAGGCTACCCGAAGGTGGG<br>AATATTGGGGGAGAAAAGGTGACAGGCGTTTGTTCTCTCCTCCGGCTATTCTAAACT<br>CCCACGGTTGGGTAGGTGAAGGAAGGAAAGCTAAAGAAAAAAAAGGAATTTTTGTC<br>TAATGTAAGATTTTCTTTTCTTCTTTGATTTTTTACACCCAAAAAAAAAATTTCTTATTAA<br>AAAAGATTTGAGTTTTCCTTTTTTATATTTCAGAATTAATTCAAGTCATATATACAGCAA<br>AAAACCAAATACAAAAATGCAGATTTTCGTCAAGACTTTGACCGGTAAAACCATAACA<br>TTGGAAGTTGAATCTTCCGATACCATCGACAACGTTAAGTCGAAAATT
terminator_ TPM1000_09	GATCCCAACGAAAAGAGAGACCACATGGTCCTTCTTGAGTTTGTAACAGCTGCTGG<br>GATTACACATGGCATGGATGAACTATACAAATAGATAATCTATGTAAAAGATTTATATCT<br>AAATATAATATCTTTAACACGTATACACAAGTTACCGATCACGTGGGGATTCTTGGGA<br>ATTGAAAAAAGTGTTTGCGCTTATGCAAGCCGCTTAGGTAAAAGGTCACCCATTATAT<br>ATGTATTAACTAAATTCATGGAATTAATCATGAAACCCAAAAACAATCAACTAAGAAAA<br>AAACAAAACAGAAAAACCGGAGTTTTGGATGCCAATTTTTTCTATTTTGATCCACACG<br>ACTAGCGCTTTCAGATATTAAAAAGTTTAGATGTAGGTTTTAGCGGTAACAGTTATATA<br>AATCGTGTTTCTTCTCTTGATGAAA
promoter_ TPM1000_10	TTTAGCACGCGGGGTGTAACTCAACAGAAAAATGTGCCATAGAACAAGACTAGGCA<br>AAAGCCAAGGAGCGTTTGCCATGAACTTCCACAATGGAACGAGAAAGCATGTGGTC<br>TTGAACGGTTCCTGATCCTATAATCTAAGAACATTAATTTACTCATTCACCTACTTCCA<br>CCAGTAATACCACAGGTACATATACCCTATCAACGACCTACATCATCGTAGGGTCAAT<br>GGGAGTCTGCAACGCGGTCTACCCTCCAGGACAGAACCCCACCTGATCGAGGGG<br>ATAGTCGCTCACGCGTTACCACAGCGGCTATGTACATTTGTCCAGTAAGGTTCTTGT<br>ACCTTCATCCGACACCACTCACCCCCTGTTGCTTTCTCCTTGCTGCGTCACAATTTT<br>CCCCGCGGCGACTCAATGAGTGAATTAAATTGAAAATAAACTCATGTATTTTCTAGGT<br>TTTCTTTATTCCTCTTTTGTATTGTTAAATTATTTTTCCTTTTTTTCTTCCTCTAATCATT<br>TATATTGAAAAGTATTTCATTCAAGTGAGATTAACTCAACACAAAAACAAAACTATTATA<br>TATTACACTAAAAAATGCAGATTTTCGTCAAGACTTTGACCGGTAAAACCATAACATT<br>GGAAGTTGAATCTTCCGATACCATCGACAACGTTAAGTCGAAAATT
terminator_ TPM1000_10	GATCCCAACGAAAAGAGAGACCACATGGTCCTTCTTGAGTTTGTAACAGCTGCTGG<br>GATTACACATGGCATGGATGAACTATACAAATAGAGGATTATATATACTTTGTATTATAG<br>TTAAAATAAGTAAGTCATTGAACACCTTAGTTTATTACTATTCGCTGGTCCTCTACTGA<br>TTGTCATAGTGACTTTTATGTAATGGAAAGCATTTGCCATTCAACAGTATAAACGCTAA<br>TCTCTAGAAAAACAGACACTTTCATCGAGTAAAGGTAGAGGCACTTATAAATACACAA<br>CCATTAAAGCCGATTATGAAACATGATAAAGGCACTACCTGATCCCTAACTACCAACT<br>CCCTCTATTTTTTTACACCAAACACACACGACTAGCGCTTTCAGATATTAAAAAGTTTA<br>GATGTAGGTTTTAGCGGTAACAGTTATATAAATCGTGTTTCTTCTCTTGATGAAA

Promoter_RPL3 and terminator_RPL3 have 40 bp homologous sequences flanking both ends for ligation with GFP and integration into XI-2 genomic site. Other promoters and terminators have 90 bp homologous sequences flanking both ends.

Supplementary Table 8. List of PCR primers.

primer name	sequence
L90	TTTAGCACGCGGGGTGTAA
R90	TTTCATCAAGAGAAGAAACACG
promoter_YOR063W_fwd	TAGGCAAAAGCCAAGGAGCGTTTGCCATGAACTTCCACAATTATTAAA TTCAGTGGTAATGCAA
promoter_YOR063W_rev	TTATGGTTTTACCGGTCAAAGTCTTGACGAAAATCTGCATTGATTGATT GTTGTAGTAACTGTG
terminator_YOR063W_fwd	TGCTGGGATTACACATGGCATGGATGAACTATACAAATAGAGAAGTTT TGTTAGAAAATAAATCATTTTT
terminator_YOR063W_rev	ACATCTAAACTTTTTAATATCTGAAAGCGCTAGTCGTGTGGGCTTGTCC CTTCGAGTG
pUC19_fwd	TGTTTCTTCTCTTGATGAAAGGGTACCGAGCTCGAATTC
pUC19_rev	GTTACACCCCGCGTGCTAAAGGGGATCCTCTAGAGTCG
909	GTTTGTAGTTGGCGGTGGAG
910	GAGACAAGATGGGGCAAGAC
GFP_rev	AATTTTCGACTTAACGTTGTCG
GFP_fwd	GATCCCAACGAAAAGAGAG
GFP_qPCR_fwd	GTCACTACTCTCACTTATGGTGTTC
GFP_qPCR_rev	GTGTCTTGTAGTTCCCGTCATC
TAF10_qPCR _fwd	ATATTCCAGGATCAGGTCTTCCGTAGC
TAF10_qPCR _rev	GTAGTCTTCTCATTCTGTTGATGTTGTTGTTG

Supplementary Table 9. List of DNA fragments used in constructs for the mutagenesis experiment.

construct	sequence
*UBIMΔkGFP**	ATGCAGATTTTCGTCAAGACTTTGACCGGTAAAACCATAACATTGGAAGTTGAATCTTCC<br>GATACCATCGACAACGTTAAGTCGAAAATTCAAGACAAGGAAGGTATCCCTCCAGATCAA<br>CAAAGATTGATCTTTGCCGGTAAGCAGCTAGAAGACGGTAGAACGCTGTCTGATTACAA<br>CATTCAGAAGGAGTCCACCTTACATCTTGTGCTAAGGCTAAGAGGTGGTATGCACGGAT<br>CCGGAGCTTGGCTGTTGCCCGTCTCACTGGTGAAAAGAAAAACCACCCTGGCGCCCAA<br>TACGAGTAAAGGAGAAGAACTTTTCACTGGAGTTGTCCCAATTCTTGTTGAATTAGATGG<br>TGATGTTAATGGGCACAAATTTTCTGTCAGTGGAGAGGGTGAAGGTGATGCAACATACG<br>GAAAACTTACCCTTAAATTTATTTGCACTACTGGAAAACTACCTGTTCCATGGCCAACACT<br>TGTCACTACTCTCACTTATGGTGTTCAATGCTTTTCAAGATACCCAGATCACATGAAACAG<br>CATGACTTTTTCAAGAGTGCCATGCCCGAAGGTTATGTACAGGAAAGAACTATATTTTTCA<br>AAGATGACGGGAACTACAAGACACGTGCTGAAGTCAAGTTTGAAGGTGATACCCTTGTT<br>AATAGAATCGAGTTAAAAGGTATTGATTTTAAAGAAGATGGAAACATTCTTGGACACAAAT<br>TGGAATACAACTATAACTCACACAATGTATACATCATGGCAGACAAACAAAAGAATGGAAT<br>CAAAGCTAACTTCAAAATTAGACACAACATTGAAGATGGAAGCGTTCAACTAGCAGACCA<br>TTATCAACAAAATACTCCAATTGGCGATGGCCCTGTCCTTTTACCAGACAACCATTACCTG<br>TCCACACAATCTGCCCTTTCGAAAGATCCCAACGAAAAGAGAGACCACATGGTCCTTCT<br>TGAGTTTGTAACAGCTGCTGGGATTACACATGGCATGGATGAACTATACAAATAG
promoter_POP6	TTTAGCACGCGGGGTGTAACTCAACAGAAAAATGTGCCATAGAACAAGACTAGGCAAAA<br>GCCAAGGAGCGTTTGCCATGAACTTCCACAATCTCTTGATTATGTCATATGAAAGGTCCA<br>GTGGGACTTGCTTTTGTTGCAGCACCTTTGCTAATGAATGAAAGGCACATAGTGACTGCT<br>TAAAAATGCAGGAACTTAAATTATTCCGAATGGTATTTTGTCTCACATATATTGTCCCATAC<br>TGTGCCAGGATCCCGGCTTTACCCAGTATCATCATTGTACCGTTACCAATTCTCCTCGTAT<br>ATCACGGTTAGTTTTTAAACCTCGGGGTGACGTTTACTATTGGCGTACTAATATATTCTTAT<br>TTTCTTTTCTTTTTTGTTGGCAGTTTCAAGCAACACATGTACTGGATAACCAACCCCCGC<br>ACGCTCTTGGAAAAAATTGAGAAGGCATCGGACACTTGCTGATGAGTATTTCGAAAAATT<br>CCATGAAAGATGAGGCCAAGATTGTTTGGAAGAGATTGAAAAGAAGAAGAAGAAAAAAA<br>GATAAAAGCAAATCAAAAATGCAGATTTTCGTCAAGACTTTGACCGGTAAAACCATAACAT<br>TGGAAGTTGAATCTTCCGATACCATCGACAACGTTAAGTCGAAAATT
terminator_POP6	GATCCCAACGAAAAGAGAGACCACATGGTCCTTCTTGAGTTTGTAACAGCTGCTGGGAT<br>TACACATGGCATGGATGAACTATACAAATAGAATCGACCAGCTCTTTTAGCATCCATAGCT<br>ACTTCTTGCATTTGTACTTTATAATATAAAGCATTTTTAGAAGGCTTAATCGATATCAGAACT<br>ACCAATTGTTCCTACTACAAGAAGTATGTGTCATTGAATGAAAGAAAAAAGGATGCATGG<br>AAATAGCACAACTTTATTTATTTTCTTCCCTTTGGAAATCGGAAATTCAATGATATGCCCTC<br>TGATATAATAGCAAAGACATGATCGTTAATTTAGGCCTCGCTTTGAATCCCACACGACTAG<br>CGCTTTCAGATATTAAAAAGTTTAGATGTAGGTTTTAGCGGTAACAGTTATATAAATCGTGT<br>TTCTTCTCTTGATGAAA
promoter_RPL3	TAGGCAAAAGCCAAGGAGCGTTTGCCATGAACTTCCACAATTATTAAATTCAGTGGTAAT<br>GCAACAGCAAGAGGAAAGGTGGAGGGATTAACGCATTTCAGACAGCTTTATAGGGGGA<br>AAGAAAGCACTCGCAAACTTGCTGCCTGTTCGCAGTCATTGGTTGCAAAAACTAAACTC<br>TACTCACGCACACTGGAATGAATGGCAATATTCTTTTTTAGGTTAACCGGCCGGACAGTA<br>ATATAGTAATCGTTTTGTACGTTTTTCAAGAAGCGACGCACAACTGTTTTCCATTTTTTTTT<br>TTTTTTTTTCAGTGATCATCGTCCATGAAAAAAATTTTTCATTTGTCTCTTTCGTGCTTCCT<br>GGATATATAAAATACGATTTATTTAGTTGTCTTTGTCAATCCTCATCTTTCTTTACTCATTATT<br>TCATTTCGGTTTTGTTCATCTCTAGAACAACACAGTTACTACAACAATCAATCAATGCAGA<br>TTTTCGTCAAGACTTTGACCGGTAAAACCATAA

terminator_RPL3	TGCTGGGATTACACATGGCATGGATGAACTATACAAATAGAGAAGTTTTGTTAGAAAATAA<br>ATCATTTTTTAATTGAGCATTCTTATTCCTATTTTATTTAAATAGTTTTATGTATTGTTAGCTAC<br>ATACAACAGTTTAAATCAAATTTTCTTTTTCCCAAAGTCCAAAATGGAGGTTTATTTTGATG<br>ACCCGCATGCGATTATGTTTTGAAAGTATAAGACTACATACATGTACATATATTTAAACATGT<br>AAACCCGTCCATTATATTGCTTACTTTCTTCTTTTTTGCCGTTTTGACTTGGACCTCTGGT<br>TTGCTATTTCCTTACAATCTTTGCTACAATACCATTTGCCCTTGGGAGCTTGTTTCAGGCC<br>TACGCAACCATAATGGAACCACTCGAAGGGACAAGCCCACACGACTAGCGCTTTCAGAT<br>ATTAAAAAGTTTAGATGT
promRPL3_variant<br>3000527	TTTAGCACGCGGGGTGTAACTCAACAGAAAAATGTGCCATAGAACAAGACTAGGCAAAA<br>GCCAAGGAGCGTTTGCCATGAACTTCCACAATTATTAAATTCAGTGGTAATGCAACAGCA<br>AGAGGAAAGGTGGAGGGATTAACGCATTTCAGACAGCTTTATAGGGGGAAAGAAAGCAC<br>TCGCAAACTTGCTGCCTGTTCGCAGTCATTGGTTGCAAAAACTAAACTCTACTCACGCAC<br>ACTGGAATGAATGGCAATATTCTTTTTTAGGTTAACCGGCCGGACAGTAATATAGTAATCG<br>TTTTGTACGTTTTTCAAGAAGCGACGCACAACTGTTTTCCATTTTTTTTTTTTTTAATTTCAG<br>TGATCATCGTCCATGAATAAAATTTTTGATTTGTCTTATTCGTGCTTCCTGGATATATAAAAT<br>ACGATTACTTTAGTTATCTTCGTTAATCCCCATCTATCTATACTCATCATCACACTTCGGAT<br>GTGTTCATCTCTAGAACAACACAGTTACTACAACAATCAATCAATGCAGATTTTCGTCAAG<br>ACTTTGACCGGTAAAACCATAACATTGGAAGTTGAATCTTCCGATACCATCGACAACGTT<br>AAGTCGAAAATT
promRPL3_variant<br>3059399	TTTAGCACGCGGGGTGTAACTCAACAGAAAAATGTGCCATAGAACAAGACTAGGCAAAA<br>GCCAAGGAGCGTTTGCCATGAACTTCCACAATTATTAAATTCAGTGGTAATGCAACAGCA<br>AGAGGAAAGGTGGAGGGATTAACGCATTTCAGACAGCTTTATAGGGGGAAAGAAAGCAC<br>TCGCAAACTTGCTGCCTGTTCGCAGTCATTGGTTGCAAAAACTAAACTCTACTCACGCAC<br>ACTGGAATGAATGGCAATATTCTTTTTTAGGTTAACCGGCCGGACAGTAATATAGTAATCG<br>TTTTGTACGTTTTTCAAGAAGCGACGCACAACTGTTTTCCATTTTTTTTTTTCGTTTTTTCA<br>GTGATCATCGTCCATGAAAAAAATCTGTCATTGCACTCTTTCGTGCTTCCTGGATATATAA<br>AATACGAGTTATTTAGTTGTATTTGTCAAGTTTCTTCCTTACTTTCTTATTATTTTATTTCCGT<br>TTTGTTCATCTCTAGAACAACACAGTTACTACAACAATCAATCAATGCAGATTTTCGTCAA<br>GACTTTGACCGGTAAAACCATAACATTGGAAGTTGAATCTTCCGATACCATCGACAACGT<br>TAAGTCGAAAATT
promRPL3_variant<br>3080857	TTTAGCACGCGGGGTGTAACTCAACAGAAAAATGTGCCATAGAACAAGACTAGGCAAAA<br>GCCAAGGAGCGTTTGCCATGAACTTCCACAATTATTAAATTCAGTGGTAATGCAACAGCA<br>AGAGGAAAGGTGGAGGGATTAACGCATTTCAGACAGCTTTATAGGGGGAAAGAAAGCAC<br>TCGCAAACTTGCTGCCTGTTCGCAGTCATTGGTTGCAAAAACTAAACTCTACTCACGCAC<br>ACTGGAATGAATGGCAATATTCTTTTTTAGGTTAACCGGCCGGACAGTAATATAGTAATCG<br>TTTTGTACGTTTTTCAAGAAGCGACGCACAACTGTTTTCCATTTTTTTTTTTTTTGTTGGCA<br>GTGATCATCGTCCATGAAAAAAGTTTTTCGTAAGTATCTTGCGTGCTTCCTGGATATATAA<br>AATACGTTTTATTTAGGTGGCTATGTCAATCCTCTTCCTTCTTTCCTCGTTCTTTCTTTTCC<br>GTTTTGTTCATCTCTAGAACAACACAGTTACTACAACAATCAATCAATGCAGATTTTCGTC<br>AAGACTTTGACCGGTAAAACCATAACATTGGAAGTTGAATCTTCCGATACCATCGACAAC<br>GTTAAGTCGAAAATT
promRPL3_variant<br>3438152	TTTAGCACGCGGGGTGTAACTCAACAGAAAAATGTGCCATAGAACAAGACTAGGCAAAA<br>GCCAAGGAGCGTTTGCCATGAACTTCCACAATTATTAAATTCAGTGGTAATGCAACAGCA<br>AGAGGAAAGGTGGAGGGATTAACGCATTTCAGACAGCTTTATAGGGGGAAAGAAAGCAC<br>TCGCAAACTTGCTGCCTGTTCGCAGTCATTGGTTGCAAAAACTAAACTCTACTCACGCAC<br>ACTGGAATGAATGGCAATATTCTTTTTTAGGTTAACCGGCCGGACAGTAATATAGTAATCG<br>TTTTGTACGTTTTTCAAGAAGCGACGCACAACTGTTTTCCATTTTTTTTTTGCTTCTTCACA<br>GTGATCATCGTCCATGAATAACCCTGAGCATGCGTCGTGTTCGTGCTTCCTGGATATATA<br>AAATACTAATAGGTGAGTTTTATATGACAAGCTTAATTGTTCTTTTCTCTTTGTTTCATTGAT<br>TTATTGTTCATCTCTAGAACAACACAGTTACTACAACAATCAATCAATGCAGATTTTCGTCA<br>AGACTTTGACCGGTAAAACCATAACATTGGAAGTTGAATCTTCCGATACCATCGACAACG<br>TTAAGTCGAAAATT

promRPL3_variant 3480767	TTTAGCACGCGGGGTGTAACTCAACAGAAAAATGTGCCATAGAACAAGACTAGGCAAAA<br>GCCAAGGAGCGTTTGCCATGAACTTCCACAATTATTAAATTCAGTGGTAATGCAACAGCA<br>AGAGGAAAGGTGGAGGGATTAACGCATTTCAGACAGCTTTATAGGGGGAAAGAAAGCAC<br>TCGCAAACTTGCTGCCTGTTCGCAGTCATTGGTTGCAAAAACTAAACTCTACTCACGCAC<br>ACTGGAATGAATGGCAATATTCTTTTTTAGGTTAACCGGCCGGACAGTAATATAGTAATCG<br>TTTTGTACGTTTTTCAAGAAGCGACGCACAACTGTTTTCCATTTTTTTTTTTCAATAGTCA<br>GTGATCATCGTCCATGAAGGACATCAGCCAGGTGGCTCTGTCGTGCTTCCTGGATATATA<br>AAATATCTTTTCTTGAGTTGAATTTGTACATTGTTTTCGATCGTTACTTATTCCTTCCGTTCT<br>TATTTGTTCATCTCTAGAACAACACAGTTACTACAACAATCAATCAATGCAGATTTTCGTCA<br>AGACTTTGACCGGTAAAACCATAACATTGGAAGTTGAATCTTCCGATACCATCGACAACG<br>TTAAGTCGAAAATT
promRPL3_variant 3529331	TTTAGCACGCGGGGTGTAACTCAACAGAAAAATGTGCCATAGAACAAGACTAGGCAAAA<br>GCCAAGGAGCGTTTGCCATGAACTTCCACAATTATTAAATTCAGTGGTAATGCAACAGCA<br>AGAGGAAAGGTGGAGGGATTAACGCATTTCAGACAGCTTTATAGGGGGAAAGAAAGCAC<br>TCGCAAACTTGCTGCCTGTTCGCAGTCATTGGTTGCAAAAACTAAACTCTACTCACGCAC<br>ACTGGAATGAATGGCAATATTCTTTTTTAGGTTAACCGGCCGGACAGTAATATAGTAATCG<br>TTTTGTACGTTTTTCAAGAAGCGACGCACAACTGTTTTCCATTTTTTTTTGCTTTTTCTCA<br>GTGATCATCGTCCATGAAACTATTTTTTTTATTGATTTTTTCGTGCTTCCTGGATATATAAAA<br>TAGGCTCAATTTAGTTTATTTGGACAATCAGCATCGTAATCAGCTCCTATAAAAATAGCGG<br>AATTGTTCATCTCTAGAACAACACAGTTACTACAACAATCAATCAATGCAGATTTTCGTCA<br>AGACTTTGACCGGTAAAACCATAACATTGGAAGTTGAATCTTCCGATACCATCGACAACG<br>TTAAGTCGAAAATT
promRPL3_variant 7861215	TTTAGCACGCGGGGTGTAACTCAACAGAAAAATGTGCCATAGAACAAGACTAGGCAAAA<br>GCCAAGGAGCGTTTGCCATGAACTTCCACAATTATTAAATTCAGTGGTAATGCAACAGCA<br>AGAGGAAAGATGGGGCGATTAACGCATTTCAGACAGCTTTATAGGGGGAAAGAAAGCAC<br>TCGCAAACTTGCTGCCTGTTCACAGTCATTGGTTGCAAAAACTAAACTCTACTCTCGCTC<br>ACTGGAATGAATGGCAATATTCTTTTTTAGGTTAACCGGCAGGATAGTAATATAGTAATCGT<br>TTTGTACGTTTTTCAAGAAGCGACGCACAACTGTTTTCCATTATTTTTTTGTTTTTTTCAGT<br>GATCATCGTCCATTAAAAAAATTTTTCATTTGTCTCTTTAGTGCTTCCTGGATATATAAAATA<br>CGATTGATTTAATTGTCTTTGACAATCCTCATCTTCCTTTAATCATCGATTCATTTCGGTTT<br>TGTTCATCTCTAGAACAACACAGTTACTACAACAATCAATCAATGCAGATTTTCGTCAAGA<br>CTTTGACCGGTAAAACCATAACATTGGAAGTTGAATCTTCCGATACCATCGACAACGTTA<br>AGTCGAAAATT
promRPL3_variant 8172100	TTTAGCACGCGGGGTGTAACTCAACAGAAAAATGTGCCATAGAACAAGACTAGGCAAAA<br>GCCAAGGAGCGTTTGCCATGAACTTCCACAATGATTAAATTCAGGGGTAATGCAGCAGC<br>AAGAGGAAAGGTGGGGCGATTAACCCATTTCAGACAGCCTTATAGGGGGAAGGAAAGC<br>ACTCGCAATCTTGCTGCCTTTTCGCTGTAATTGGTTGCAAAAACTAGACTCTACTCACGC<br>ACACTGGAATGAATGTGAATATTCTTTTTTAGGTCAACAGGCCGGACAGTAAAATAGTAAT<br>TGTTTTGGACGTTTTTCAAGAAGCGACGCACAACTGTTTTCCATTTTTTTCTTTTTTTTTA<br>AAGTGATCATACTCCATGAAAAAAATGTTTAATTTGTCTCTTTCGTGCTACCTGGATATATA<br>AAATACGATCACTTTAGTTTTCTTTGCCCATCCTCATCTATCACTTCTCATTATATCATTTCG<br>GTATTGTTCATCTCTAGAACAACACAGTTACTACAACAATCAATCAATGCAGATTTTCGTC<br>AAGACTTTGACCGGTAAAACCATAACATTGGAAGTTGAATCTTCCGATACCATCGACAAC<br>GTTAAGTCGAAAATT
promRPL3_variant 8207535	TTTAGCACGCGGGGTGTAACTCAACAGAAAAATGTGCCATAGAACAAGACTAGGCAAAA<br>GCCAAGGAGCGTTTGCCATGAACTTCCACAATTATTAAATTCAGTGGTAATGCAAGAGTA<br>AGAGGAGAGGTGGGGGGATTAACGCATTTCAGACAGCTTTATAGGAGGAAAGAAAGGAT<br>TCGCGGACTTGCTGGCTGTTCGTAGTCTATGGCTGCAAAAACTACACTCTACTCACGCAT<br>ACTGGAATGGTTGGCAATATTCTTTTTTAGGTTAACCGGCCGGACAGTAATATAGTAATCG<br>TTTTGTACGTTTCTCAAGAAGAGACGAACAACTGTTTTGCATTTATTTTTTTTTCTTTTCCG<br>TGATCGTCGACCATTAAAAAAATTTTTCATTCGTCGCTTGCGTGCTTCCTGGATATATAAA<br>ACACGAGTTATTTAGTTGTCTTTGTCAATTCTCAACTTTCTTTATTCATTATTTCAATTCCGT<br>TCTTTTCATCTCTAGAACAACACAGTTACTACAACAATCAATCAATGCAGATTTTCGTCAA<br>GACTTTGACCGGTAAAACCATAACATTGGAAGTTGAATCTTCCGATACCATCGACAACGT<br>TAAGTCGAAAATT

promRPL3_variant 8248101	TTTAGCACGCGGGGTGTAACTCAACAGAAAAATGTGCCATAGAACAAGACTAGGCAAAAGCCAAGGAGCGTTTGCCATGAACTTCCACAATTATTAAATTCAGTGGTAATGCAAAAGCAAGGGGAAAGGTGGAGGGATAAACGCATTTCCGACGGCTTTATAGGCGGAAAGAAAGCACTCACAGACTTGCGGCCTGTTCACAGTCATTCGTTGCAAAAACAAAACTCTACTCACGCACACTGGAATGAATGGCAATATTCTTCTTTAGGTTTACCGGCCGGACAGTAATATATTAATCGTTTCGTACGTTTTCAAAGAAGCGACGCACAACTGTTTTCCATTTTTATTTTTTTCTCTTCGGTGATCATCGTCCTTGAAAAAATTTTTTCAGCTGCCTCTTTCGTGCTTCCTGGATATATAAAATACGATTTATTTAGTTGTATTTTTCAGGCCTCATTCTTCATTCCTTGTTATTTCATTTCGTTTTTCCTCATCTCTAGAACAACACAGTTACTACAACAATCAATCAATGCAGATTTTCGTCAAGACTTTGACCGGTAAAACCATAACATTGGAAGTTGAATCTTCCGATACCATCGACAACGTTAAGTCGAAAATT

Supplementary Table 10. Minimal Media Recipe.

	Minimal Media
KH_2PO_4	14.4 g·L^{-1}
$MgSO_4 \cdot 7H_2O$	0.5 g·L^{-1}
$(NH_4)SO_4$	7.5 g·L^{-1}
Trace metals stock solution*	2 ml·L^{-1}
Vitamin stock solution**	1 ml·L^{-1}
Glucose 50%	40 mL·L^{-1}

*Trace metals solution: $FeSO_4$•7H_2O 3.0 g·L^{-1}, $ZnSO_4$•7H_2O 4.5 g·L^{-1}, $CaCl_2$•2H_2O 4.5 g·L^{-1}, $MnCl_2$•4H_2O 1 g·L^{-1}, $CoCl_2$•6H_2O 300 mg·L^{-1}, $CuSO_4$•5H_2O 300 mg·L^{-1}, Na_2MoO_4•2H_2O 400 mg·L^{-1}, H_3BO_3 1 g·L^{-1}, KI 100 mg·L^{-1}, Na_2EDTA•2H_20 19 g·L^{-1}

**Vitamins solution: d-Biotin 50 mg·L^{-1}, D-Pantothenic acid hemicalcium salt 1.0 g·L^{-1}, Thiamin-HCl 1.0 g·L^{-1}, Pyridoxin-HCl 1.0 g·L^{-1}, Nicotinic acid 1.0 g·L^{-1}, 4-aminobenzoic acid 0.2 g·L^{-1}, myo-Inositol 25 g·L^{-1}

The pH of the media was adjusted to 6.5 using KOH pellets. After sterilization, 2 mL·L^{-1} of the trace element solution and 1 mL·L^{-1} of the vitamin solution were added.

Supplementary Table 11. Overview of data sources used in the study.

Description	Link
Saccharomyces cerevisiae S288c R64-1-1 ORFs, UTRs	ftp://ftp.ensemblgenomes.org/pub/fungi/release-41/gff3/saccharomyces_cerevisiae
Saccharomyces cerevisiae S288c R64-1-1 genome sequence	ftp://ftp.ensemblgenomes.org/pub/fungi/release-41/fasta/saccharomyces_cerevisiae/dna/
Saccharomyces cerevisiae S288c R64-1-1 additional Xu et al. 2009 UTRs [28]	https://downloads.yeastgenome.org/published_datasets/Xu_2009_PMID_19169243/track_files/Xu_2009_ORF-Ts_V64.gff3
Saccharomyces cerevisiae S288c R64-1-1 additional Nagalakshmi et al. 2008 UTRs [29]	https://science.sciencemag.org/highwire/filestream/589738/field_highwire_adjunct_files/1/1158441_tables_s2_to_s6.zip
SGD gene names [30]	https://downloads.yeastgenome.org/curation/chromosomal_feature/SGD_features.tab
SGD motif information [30]	https://www.yeastgenome.org
Transcriptomics data [31]	http://dee2.io/mx/
Yeast Jaspar DNA seq motifs (JASPAR2018_CORE_fungi_non-redundant.meme) [1]	http://meme-suite.org/meme-software/Databases/motifs/motif_databases.12.19.tgz
Yeastract DNA seq motifs (YEASTRACT_20130918.meme) [27]	http://meme-suite.org/meme-software/Databases/motifs/motif_databases.12.19.tgz
Deep learning-uncovered expression-related motifs and motif associations [8]	https://zenodo.org/record/3905252
80 bp proximal promoter designs [32]	https://zenodo.org/record/4436477
5' UTR designs [14]	https://github.com/Seeliglab/2017---Deep-learning-yeast-UTRs/tree/master/Data

Supplementary References

1. Khan, A. *et al.* JASPAR 2018: update of the open-access database of transcription factor binding profiles and its web framework. *Nucleic Acids Res.* **46**, D1284 (2018).
2. Basehoar, A. D., Zanton, S. J. & Pugh, B. F. Identification and distinct regulation of yeast TATA box-containing genes. *Cell* **116**, 699–709 (2004).
3. Lubliner, S., Keren, L. & Segal, E. Sequence features of yeast and human core promoters that are predictive of maximal promoter activity. *Nucleic Acids Res.* **41**, 5569–5581 (2013).
4. Li, J., Liang, Q., Song, W. & Marchisio, M. A. Nucleotides upstream of the Kozak sequence strongly influence gene expression in the yeast S. cerevisiae. *J. Biol. Eng.* **11**, 25 (2017).
5. Nakagawa, S., Niimura, Y., Gojobori, T., Tanaka, H. & Miura, K.-I. Diversity of preferred nucleotide sequences around the translation initiation codon in eukaryote genomes. *Nucleic Acids Res.* **36**, 861–871 (2008).
6. Guo, Z. & Sherman, F. 3'-end-forming signals of yeast mRNA. *Trends Biochem. Sci.* **21**, 477–481 (1996).
7. Curran, K. A. *et al.* Short Synthetic Terminators for Improved Heterologous Gene Expression in Yeast. *ACS Synth. Biol.* **4**, 824–832 (2015).
8. Zrimec, J. *et al.* Deep learning suggests that gene expression is encoded in all parts of a co-evolving interacting gene regulatory structure. *Nat. Commun.* **11**, 6141 (2020).
9. Xi, L. *et al.* Predicting nucleosome positioning using a duration Hidden Markov Model. *BMC Bioinformatics* **11**, 346 (2010).
10. Kato, H., Shimizu, M. & Urano, T. Chemical map–based prediction of nucleosome positioning using the Bioconductor package nuCpos. *bioRxiv* 2019.12.25.888305 (2020) doi:10.1101/2019.12.25.888305.
11. van der Maaten, L. Visualizing Data using t-SNE. https://www.jmlr.org/papers/volume9/vandermaaten08a/vandermaaten08a.pdf?fbclid=IwAR0Bgg1eA5TFmqOZeCQXsIoL6PKrVXUFaskUKtg6yBhVXAFFvZA6yQiYx-M (2008).
12. Espinar, L., Schikora Tamarit, M. À., Domingo, J. & Carey, L. B. Promoter architecture determines cotranslational regulation of mRNA. *Genome Res.* **28**, 509–518 (2018).
13. Dvir, S., Velten, L., Sharon, E. & Zeevi, D. Deciphering the rules by which 5′-UTR sequences affect protein expression in yeast. *Proc. Natl. Acad. Sci.* **110**, E2792–E2801 (2013).
14. Cuperus, J. T., Groves, B. & Kuchina, A. Deep learning of the regulatory grammar of yeast 5′ untranslated regions from 500,000 random sequences. *Genome Res.* **27**, 1–10 (2017).
15. Cheng, J., Maier, K. C., Avsec, Ž., Rus, P. & Gagneur, J. Cis-regulatory elements explain most of the mRNA stability variation across genes in yeast. *RNA* **23**, 1648–1659 (2017).
16. Shalem, O. *et al.* Systematic dissection of the sequence determinants of gene 3'end mediated expression control. *PLoS Genet.* **11**, e1005147 (2015).
17. Morse, N. J., Gopal, M. R., Wagner, J. M. & Alper, H. S. Yeast Terminator Function Can Be Modulated and Designed on the Basis of Predictions of Nucleosome Occupancy. *ACS*

Synth. Biol. **6**, 2086–2095 (2017).
18. Zrimec, J., Buric, F., Kokina, M., Garcia, V. & Zelezniak, A. Learning the regulatory code of gene expression. *Front. Mol. Biosci.* **8**, (2021).
19. Grant, C. E., Bailey, T. L. & Noble, W. S. FIMO: scanning for occurrences of a given motif. *Bioinformatics* **27**, 1017–1018 (2011).
20. Zhao, J., Hyman, L. & Moore, C. Formation of mRNA 3' ends in eukaryotes: mechanism, regulation, and interrelationships with other steps in mRNA synthesis. *Microbiol. Mol. Biol. Rev.* **63**, 405–445 (1999).
21. Rhee, H. S. & Pugh, B. F. Genome-wide structure and organization of eukaryotic pre-initiation complexes. *Nature* **483**, 295–301 (2012).
22. Yang, C., Bolotin, E., Jiang, T., Sladek, F. M. & Martinez, E. Prevalence of the initiator over the TATA box in human and yeast genes and identification of DNA motifs enriched in human TATA-less core promoters. *Gene* **389**, 52–65 (2007).
23. Ancona, M., Ceolini, E., Öztireli, C. & Gross, M. Towards better understanding of gradient-based attribution methods for Deep Neural Networks. *arXiv [cs.LG]* (2017).
24. Neymotin, B., Ettorre, V. & Gresham, D. Multiple Transcript Properties Related to Translation Affect mRNA Degradation Rates in Saccharomyces cerevisiae. *G3* **6**, 3475–3483 (2016).
25. van Helden, J., del Olmo, M. & Pérez-Ortín, J. E. Statistical analysis of yeast genomic downstream sequences reveals putative polyadenylation signals. *Nucleic Acids Res.* **28**, 1000–1010 (2000).
26. Dvir, S. *et al.* Deciphering the rules by which 5'-UTR sequences affect protein expression in yeast. *Proc. Natl. Acad. Sci. U. S. A.* **110**, E2792–801 (2013).
27. Teixeira, M. C. *et al.* YEASTRACT: an upgraded database for the analysis of transcription regulatory networks in Saccharomyces cerevisiae. *Nucleic Acids Res.* **46**, D348–D353 (2018).
28. Xu, Z. *et al.* Bidirectional promoters generate pervasive transcription in yeast. *Nature* **457**, 1033–1037 (2009).
29. Nagalakshmi, U. *et al.* The transcriptional landscape of the yeast genome defined by RNA sequencing. *Science* **320**, 1344–1349 (2008).
30. Cherry, J. M. *et al.* SGD: Saccharomyces Genome Database. *Nucleic Acids Res.* **26**, 73–79 (1998).
31. Ziemann, M., Kaspi, A. & El-Osta, A. Digital expression explorer 2: a repository of uniformly processed RNA sequencing data. *Gigascience* **8**, 1–13 (2019).
32. Vaishnav, E. D. *et al.* The evolution, evolvability and engineering of gene regulatory DNA. *Nature* **603**, 455–463 (2022).
