# Supplementary material for: Controlling gene expression with deep generative design of regulatory DNA
Source: Nat Commun. 2022 Aug 30;13:5099. doi: 10.1038/s41467-022-32818-8 (PMC9427793; doi:10.1038/s41467-022-32818-8)
Supplement: Supplementary file 2 — Reporting Summary [file 41467_2022_32818_MOESM2_ESM.pdf]

nature portfolio

Corresponding author(s): Jan Zrimec, Aleksej Zelezniak

Last updated by author(s): 2022_08_12

Reporting Summary

Nature Portfolio wishes to improve the reproducibility of the work that we publish. This form provides structure for consistency and transparency in reporting. For further information on Nature Portfolio policies, see our Editorial Policies and the Editorial Policy Checklist.

Statistics

For all statistical analyses, confirm that the following items are present in the figure legend, table legend, main text, or Methods section.

n/a	Confirmed	
☐	☒	The exact sample size (n) for each experimental group/condition, given as a discrete number and unit of measurement
☒	☐	A statement on whether measurements were taken from distinct samples or whether the same sample was measured repeatedly
☐	☒	The statistical test(s) used AND whether they are one- or two-sided<br>*Only common tests should be described solely by name; describe more complex techniques in the Methods section.*
☒	☐	A description of all covariates tested
☐	☒	A description of any assumptions or corrections, such as tests of normality and adjustment for multiple comparisons
☐	☒	A full description of the statistical parameters including central tendency (e.g. means) or other basic estimates (e.g. regression coefficient) AND variation (e.g. standard deviation) or associated estimates of uncertainty (e.g. confidence intervals)
☐	☒	For null hypothesis testing, the test statistic (e.g. F, t, r) with confidence intervals, effect sizes, degrees of freedom and P value noted<br>*Give P values as exact values whenever suitable.*
☒	☐	For Bayesian analysis, information on the choice of priors and Markov chain Monte Carlo settings
☒	☐	For hierarchical and complex designs, identification of the appropriate level for tests and full reporting of outcomes
☐	☒	Estimates of effect sizes (e.g. Cohen's d, Pearson's r), indicating how they were calculated

Our web collection on statistics for biologists contains articles on many of the points above.

Software and code

Policy information about availability of computer code

Data collection	Python 3.6.9; Tensorflow v1.12.0; Keras v2.2.4; Hyperopt v0.1.1, IDT PrimerQuest v2.2.
Data analysis	Python 3.6.12; Tensorflow v2.4.1; Pandas 1.1.5; Scikit–learn v0.24.2; Scikit-bio v0.5.6; Scipy v1.5.4; Biopython v1.78; Fuzzywuzzy v0.18.0; Python-levenshtein v0.12.2; Seaborn v0.11.1; Meme suite v5.0.2; R v3.6; Tidyverse v1.3.0; Nucpos v3.8;<br>Code for the data analysis was deposited to the Github repository and is available at https://github.com/JanZrimec/ExpressionGAN.

For manuscripts utilizing custom algorithms or software that are central to the research but not yet described in published literature, software must be made available to editors and reviewers. We strongly encourage code deposition in a community repository (e.g. GitHub). See the Nature Portfolio guidelines for submitting code & software for further information.

Data

Policy information about availability of data

All manuscripts must include a data availability statement. This statement should provide the following information, where applicable:

- Accession codes, unique identifiers, or web links for publicly available datasets
- A description of any restrictions on data availability
- For clinical datasets or third party data, please ensure that the statement adheres to our policy

Genomic data, transcript and gene boundaries used in this study were obtained from the Saccharomyces Genome Database (https://www.yeastgenome.org/) and Ensembl (https://www.ensembl.org/), RNA sequencing data from the Digital Expression Explorer V2 database (http://dee2.io/mx/), DNA sequence motifs from the Meme suite motifs databases file (http://meme-suite.org/) and additional data from the cited references (links to raw data in Table S11). Sequence data generated in this study are provided in Tables S6, S7 and S9 and experimental data in Tables S2 and S4. Source data were deposited to the Zenodo repository and are available at https://doi.org/10.5281/zenodo.6811226.

Field-specific reporting

Please select the one below that is the best fit for your research. If you are not sure, read the appropriate sections before making your selection.

- [x] Life sciences
- [ ] Behavioural & social sciences
- [ ] Ecological, evolutionary & environmental sciences

For a reference copy of the document with all sections, see nature.com/documents/nr-reporting-summary-flat.pdf

Life sciences study design

All studies must disclose on these points even when the disclosure is negative.

Sample size	No sample size calculation was performed as no statistical distribution assumptions were made. Since deep learning models were evaluated directly on independent test sets without any assumption of underlying distributions, there was no need to rely on theoretical sample size or power calculations. Models were trained on a large variety of the publicly available RNA-Seq data and presumed to empirically capture the whole known range of gene expression variability.
Data exclusions	For gene expression levels, processed raw RNA sequencing Star counts were obtained from the Digital Expression Explorer V2 database (http://dee2.io/index.html) and filtered for experiments that passed quality control (QC tag in original database). Read counts were transformed to transcripts per million (TPM) counts and genes with zero mRNA readout (TPM < 5) were removed.
Replication	Strains were grown in biological triplicates, with experimental measurements performed in technical duplicates. All replication attempts were successful and no outliers were observed.
Randomization	Not relevant with the design of the present study as groups were not compared.
Blinding	Not relevant with the design of the present study as it does not involve clinical trials/data collection/treatment or group comparison.

Reporting for specific materials, systems and methods

We require information from authors about some types of materials, experimental systems and methods used in many studies. Here, indicate whether each material, system or method listed is relevant to your study. If you are not sure if a list item applies to your research, read the appropriate section before selecting a response.

Materials & experimental systems

n/a	Involved in the study	
☒	☐	Antibodies
☒	☐	Eukaryotic cell lines
☒	☐	Palaeontology and archaeology
☒	☐	Animals and other organisms
☒	☐	Human research participants
☒	☐	Clinical data
☒	☐	Dual use research of concern

Methods

n/a	Involved in the study	
☒	☐	ChIP-seq
☒	☐	Flow cytometry
☒	☐	MRI-based neuroimaging
